# Identification of a 10-species microbial signature of inflammatory bowel disease by machine learning and external validation

**DOI:** 10.1186/s13619-025-00246-w

**Published:** 2025-07-14

**Authors:** Shicheng Yu, Jun Li, Zhaofeng Ye, Mengxian Zhang, Xiaohua Guo, Xu Wang, Liansheng Liu, Yalong Wang, Xin Zhou, Wei Fu, Michael Q. Zhang, Ye-Guang Chen

**Affiliations:** 1 Guangzhou National Laboratory, Guangzhou, 510005 China; 2 Peking University Third Hospital, Haidian District, Beijing, 100191 China; 3 MOE Key Laboratory of Bioinformatics, School of Medicine, Tsinghua University, Beijing, 100084 China; 4 The MOE Basic Research and Innovation Center for the Targeted Therapeutics of Solid Tumors, School of Basic Medical Sciences, Nanchang University, Nanchang, 330031 China; 5 The State Key Laboratory of Membrane Biology, Tsinghua-Peking Center for Life Sciences, School of Life Sciences, Tsinghua University, Beijing, 100084 China

**Keywords:** Inflammatory bowel disease, XGBoost, Microbial species, Biomarker, Machine learning

## Abstract

Genetic and microbial factors influence inflammatory bowel disease (IBD), prompting our study on non-invasive biomarkers for enhanced diagnostic precision. Using the XGBoost algorithm and variable analysis and the published metadata, we developed the 10-species signature XGBoost classification model (XGB-IBD10). By using distinct species signatures and prior machine and deep learning models and employing standardization methods to ensure comparability between metagenomic and 16S sequencing data, we constructed classification models to assess the XGB-IBD10 precision and effectiveness. XGB-IBD10 achieved a notable accuracy of 0.8722 in testing samples. In addition, we generated metagenomic sequencing data from collected 181 stool samples to validate our findings, and the model reached an accuracy of 0.8066. The model's performance significantly improved when trained on high-quality data from the Chinese population. Furthermore, the microbiome-based model showed promise in predicting active IBD. Overall, this study identifies promising non-invasive biomarkers associated with IBD, which could greatly enhance diagnostic accuracy.

**Supplementary Information:**

The online version contains supplementary material available at 10.1186/s13619-025-00246-w.

## Background

Inflammatory bowel diseases (IBD), including ulcerative colitis (UC) and Crohn's disease (CD), have a detrimental impact on the quality of life of patients (Rutgeerts et al. 2009). Current diagnostic methods, such as clinical assessment, stool examination, endoscopy, and biopsy, face challenges owing to the nonspecific nature of symptoms associated with IBD. Distinguishing IBD from other gastrointestinal conditions with similar symptoms is complex (Liu and Chen 2020; Oliver et al. 2020; Pascal et al. 2017; Zhu et al. 2021) and difficult (Vich Vila et al. 2018). The existing therapeutic options for IBD primarily are via suppressing inflammation (Costa et al. 2005; Liu et al. 2022). Biologics such as infliximab, adalimumab, vedolizumab, and ustekinumab have been predominantly used for treating moderate-to-severe UC and CD, but their clinical effectiveness and response duration are unsatisfactory (Abu-Sbeih et al. 2020; Ferretti et al. 2023; Hyams et al. 2022; Rutgeerts et al. 2009).

Various biomarkers have been used to diagnose IBD (Yu et al. 2023). C-reactive protein (CRP) levels serve as a reliable indicator of disease progression in CD and are associated with the extent of disease in patients with UC (Henriksen et al. 2008; Vermeire et al. 2004). Fecal calprotectin has been used as an IBD marker in a subset of Spanish participants (Pascal et al. 2017). Metagenomic sequencing of species-level microbial strains, including keystone species with low abundance, plays a critical role in understanding the development of IBD (Metwaly et al. 2022). It helps identify microbial species associated with IBD, such as *Escherichia coli* (increased), *Peptostreptococcus* (increased), *Lactobacillus salivarius* (decreased), *Akkermansia* (decreased), *Roseburia* (decreased), *Fecalibacterium* (decreased), and *Clostridiales* (decreased) (Metwaly et al. 2022). Combining fecal calprotectin measurements with the top 20 taxa resulted in high prediction accuracy (area under the curve (AUC) 0.93) in a diagnostic model (Vich Vila et al. 2018), highlighting the potential of combining different markers for accurate diagnosis. Alterations in microbiome signatures are associated with disease activity, relapse risk, and response to therapy (Metwaly et al. 2022), underlining the dynamic correlation between the gut microbiome and IBD.

The Extreme Gradient Boosting (XGBoost) algorithm, a powerful machine learning ensemble method, is widely recognized for balancing prediction accuracy and interpretability (Al'Aref et al. 2020; Chen and Guestrin 2016). Its versatility makes it suitable for integrating diverse single- and multi-omics data from studies on blood pressure regulation and colon and breast cancers (Koppad et al. 2022; Louca et al. 2022; Thalor et al. 2022). The XGBoost is proficient in handling imbalanced datasets (Alam et al. 2020), and has been used for model construction based on microbiome signatures (Yuan et al. 2024). However, previous attempts to diagnose and predict IBD using machine learning models had challenges related to generalizability and varying performances across cohorts (Metwaly et al. 2022). In this study, we used variable analysis and the XGBoost algorithm to develop the 10-species signature XGBoost classification model (XGB-IBD10) for multiple IBD cohorts. To ensure comparability between metagenomic and 16S sequencing data, we employed standardization methods and constructed a machine-learning model using 70% of the samples from nine published cohorts. This model successfully identified a microbial signature involving 10 species, including well-known entities such as *Akkermansia muciniphila*. Our model outperformed existing signature, such as RISK-IBD21 (Gevers et al. 2014), UMCG IBD-IBD175 (Vich Vila et al. 2018), PRISM-IBD100 (Franzosa et al. 2019), and HMP2-IBD100 (Lloyd-Price et al. 2019), -based models, particularly when validated against our collected cohort samples. Additionally, integration of data from diverse geographical regions, including a modest amount of Chinese data, significantly augmented the predictive performance of the model.

## Results

### IBD is correlated with microbial species

**Fig. 1:**
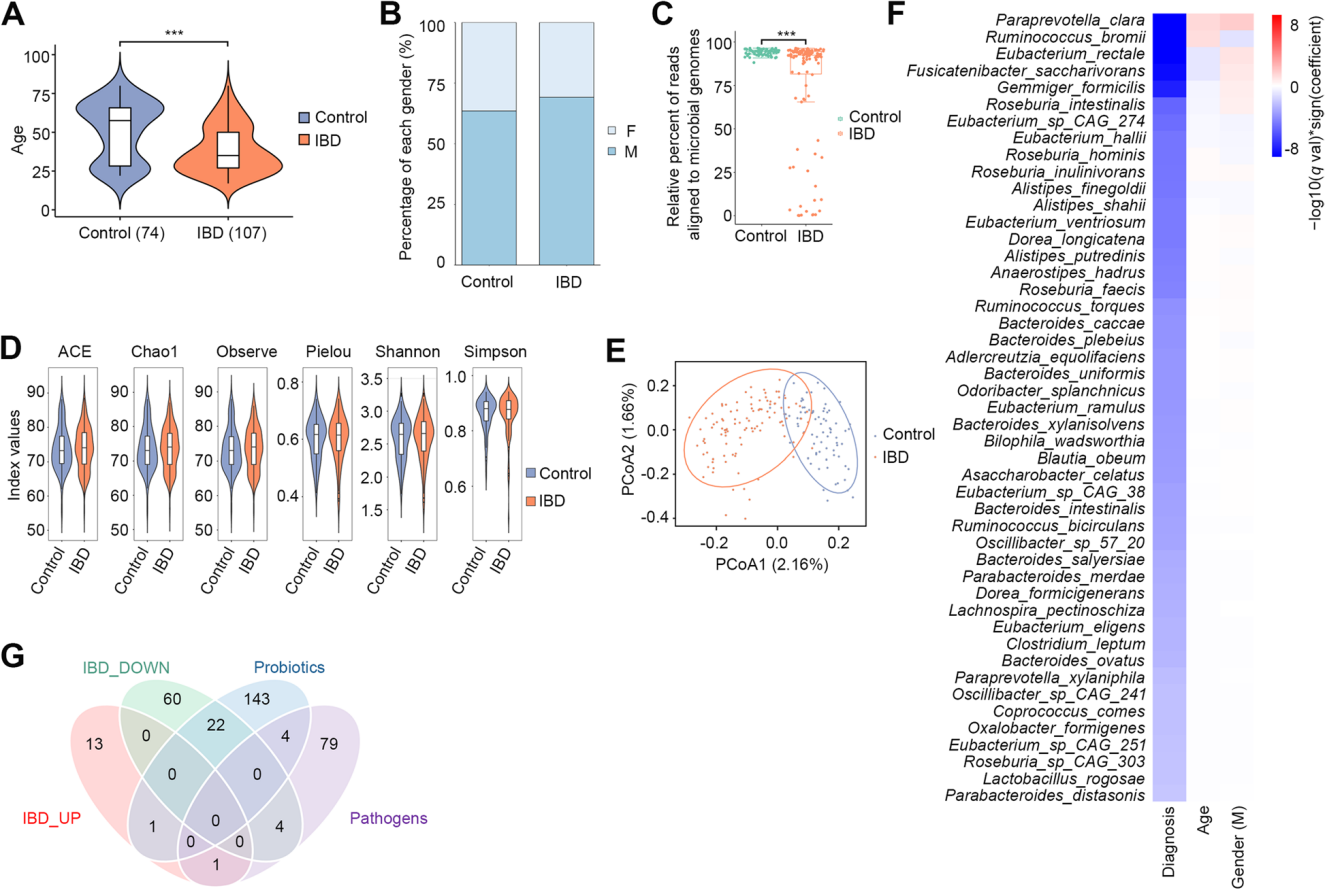
Association of IBD with microbial species. **A** The age distribution of 181 participants. **B** The gender distribution of 181 participants, **C** The percent of microbial communities of each sample. **D** The Alpha diversity indices (ACE, Chao1, Observe, Pielow, Shannon, and Simpson) of microbial communities, *p* value calculated using Mann–Whitney test. **E** The Beta diversity (PCoA) of microbial communities. **F** The correlation between microbial species and IBD classification, age, and gender. Red blocks in first column indicate a positive correlation with IBD. Red blocks in second column indicate a positive correlation with the samples come from the older groups. Red blocks in third column indicate a positive correlation with the samples come from the males. Significance for (**A**) and (**C**) is calculated by Wilcoxon rank-sum test. *p ≤ 0.05, **p < 0.01, ***p < 0.001. **G** The Venn plot shows the shared microbial species among four groups. IBD_UP: the upregulated microbial species in IBD (coefficient ≥ 1.0 and *p* value ≤ 0.05); IBD_DOWN: the downregulated microbial species in IBD (coefficient ≤−1.0 and *p* value ≤ 0.05)

In order to explore the correlation between microbial species and IBD, we collected 181 stool samples (MC-IBD cohort) from patients with IBD (*n* = 107) and healthy controls (*n* = 74). Patients with IBD underwent endoscopic examination, whereas the control group had no intestinal inflammation or discomfort. Age distribution varied significantly between the two groups (Fig. 1A), whereas sex distribution did not show notable differences (Fig. 1B). Stool samples were subjected to genomic DNA sequencing to analyze microbial species diversity. Taxonomic profiling was performed using MetaPhlAn4 (v. 4.0.2) software and the database mpa_v30_CHOCOPhlAn_201901 against reads filtered by KneadData (v. 0.10.0) software. The non-microbiome aligned read proportions were higher in the IBD samples than that in controls (Fig. 1C). Approximately 18.69% (20/107) of the IBD samples showed that over half of the sequencing reads (> 50%) were derived from non-microbiome sources, likely representing host DNA contamination. Stringent quality control measures, such as propidium monoazide treatment, have been implemented to improve aligned read proportions (Marotz et al. 2018). Alpha diversity exhibited no substantial variation between IBD and control samples across the datasets (Fig. 1D). Beta diversity analysis (principal coordinates analysis, PCoA) revealed significant differences in the microbial species composition between the IBD and control samples (Fig. 1E). Correlation analysis using Maaslin2 identified 47 microbial species significantly associated with IBD (− log10 (*q* val) * sign (coefficient) ≤ 0.01). The abundance of these microbial species was weakly correlated with age and sex than with the overall microbial species (Fig. 1F). Notably, the downregulated microbial species had a significantly higher proportion of prospectively beneficial microbial species that overlapped with previously reported probiotic data (25.58%, 22/86), in contrast to the upregulated microbial species (6.67%, 1/15) (Fig. 1G). Thus, we concluded that alterations in microbial species are critical factors in IBD. In the future, our newly gathered samples will serve as an external cohort for our model.

### XGB-IBD10 construction

**Fig. 2:**
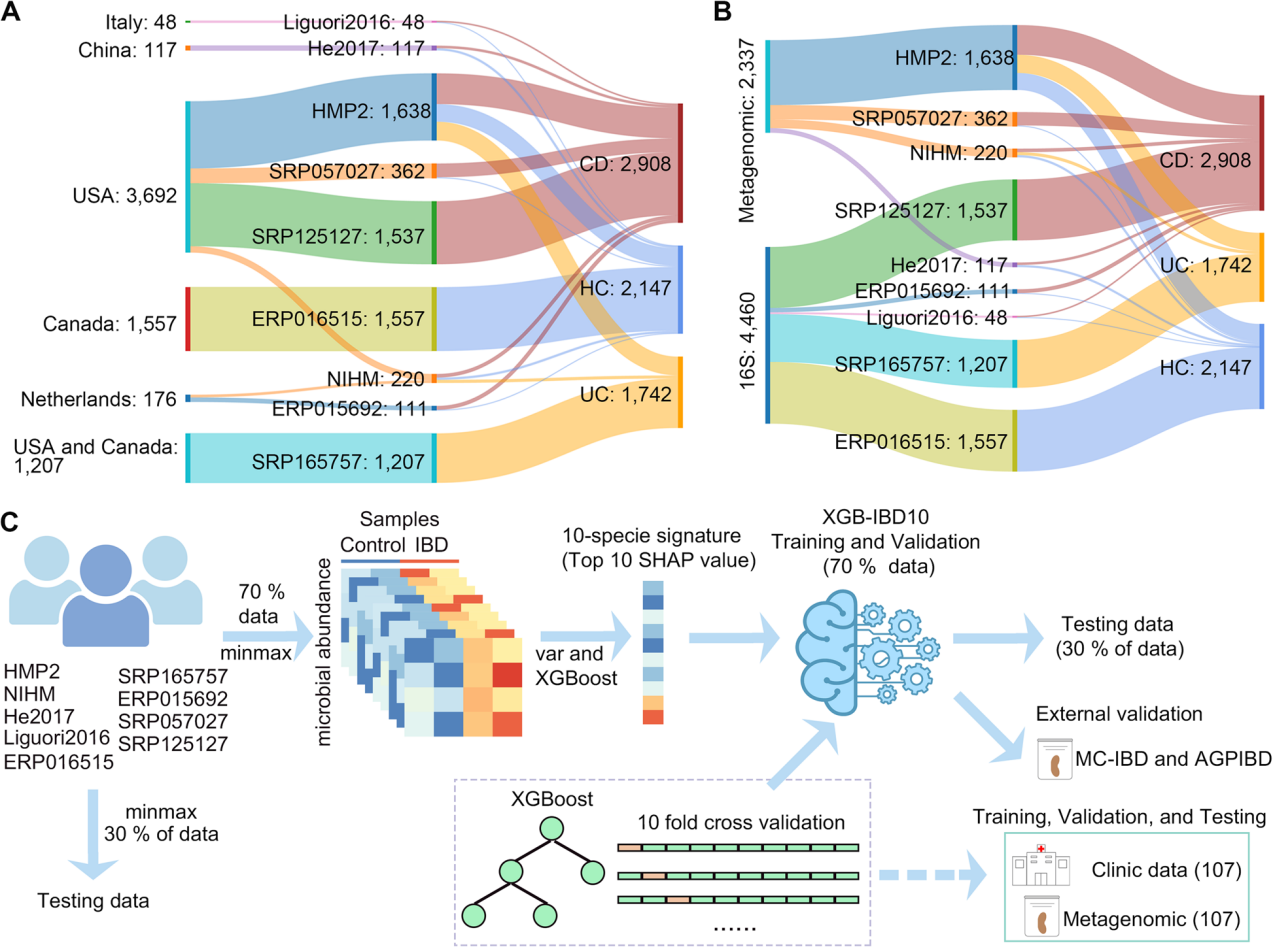
Construction of the XGBoost classification model XGB-IBD10. **A** and **B** The Schematic representation of the cohorts. **C** After IBD datasets were standardized, the var function of R and XGBoost of python was employed to select important features (10-species signature). Then, ten-fold cross-validation tests were set to compare the performance of the models between two feature sets. The XGBoost method was used to compare the performance of the XGBoost model on testing data and to predict the accuracy for the external validation data

To identify potential IBD microbial biomarkers, we employed variance component analysis, which significantly improved positive sample discrimination (Fig. 2). Specifically, SHapley Additive exPlanations (SHAP) were used to calculate the SHAP value for the top 1,000 variable species to value their importance in the model establishment (Table S1) (Lundberg and Lee 2017). All reported ‘top N’ species in subsequent analyses (e.g., top 160 species-based model) refer to species that passed both variance filtering and ranking by SHAP values. Species were selected considering the microbial genera identified in the Human Microbiome Project, NIHMS1510763, and seven cohorts (Fig. 2). Subsequently, we employed a series of standardization methods to ensure comparability between metagenomic and 16S sequencing data (see Materials and Methods for details) and constructed a machine-learning model using 70% of the samples from the nine cohorts. Using the minmax normalization method, 2,337 metagenomic samples (from the Integrative Human Microbiome Project (HMP2), NIHMS1510763, SRP057027, and He et al. 2017 cohort) and 4,460 16S samples (from SRP165757, SRP125127, ERP016515, ERP015692, and Liguori et al. 2016 cohort) were converted to the abundances of all microbial species into a range of 0 to 1 to facilitate comparison between the microbial abundances. We utilized a two-tiered strategy for data partitioning and cross-validation to enhance the validation of our predictive model. Initially, we divided the external cohorts into two subsets (allocating 70% of the data for training and reserving the remaining 30% for testing) to create a training dataset for model construction and a distinct test dataset for ultimate model assessment. Subsequently, a tenfold cross-validation was performed on 70% of the training subset data. The accuracy of the model was then evaluated using two measures: the accuracy score from the tenfold cross-validation and the accuracy score from external validation cohort. SDs were employed to quantify the variability in the performance of the model across various folds, providing valuable insights into the consistency and reliability of the model. To address the data imbalance, we utilized Synthetic Minority Over-sampling Technique (SMOTE) from imblearns to augment the number of samples in the minority class, improving the accuracy of the model (Xu et al. 2019).

**Fig. 3:**
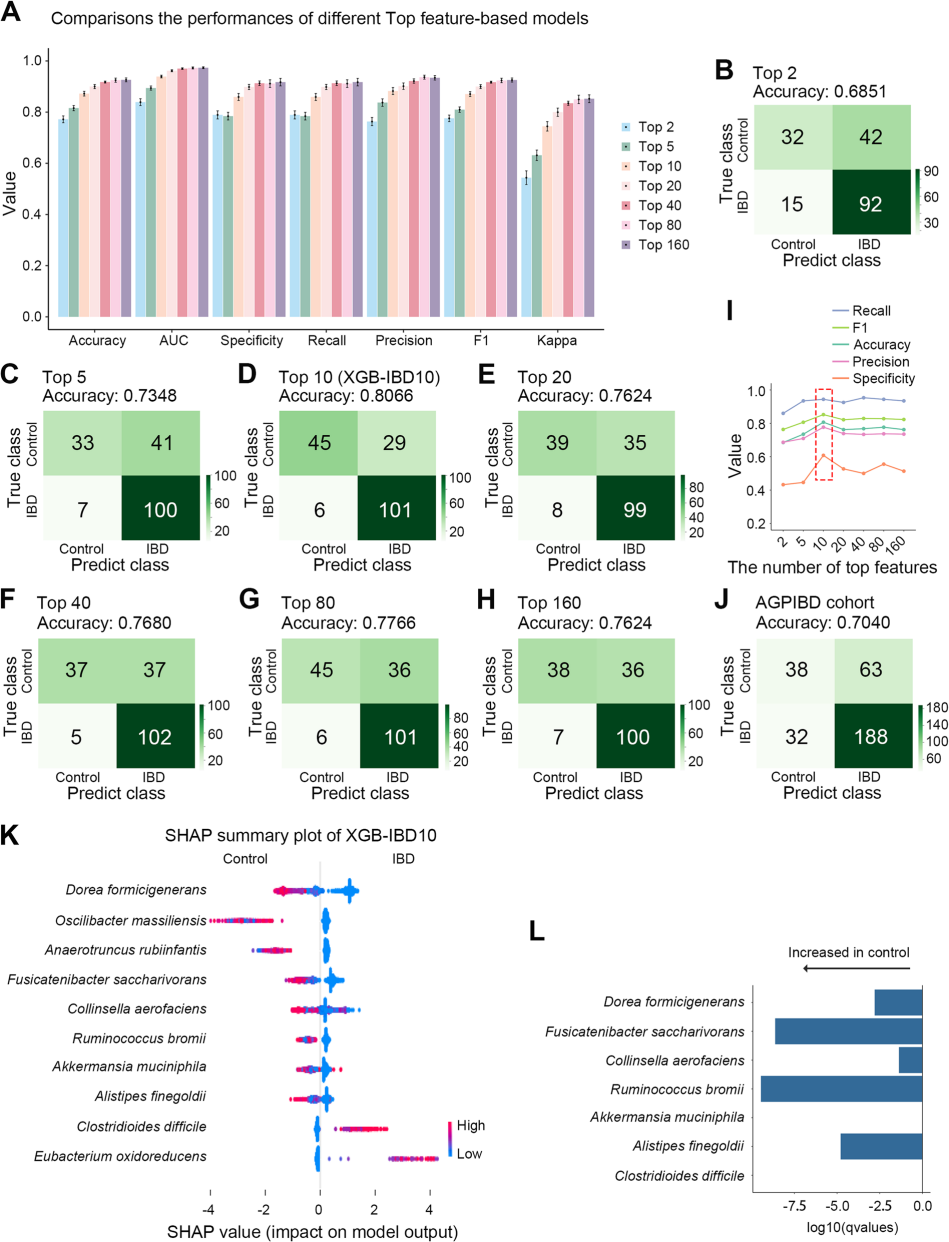
XGB-IBD10 achieves a better performance than other top species-based methods. **A** The histogram comparisons of the Accuracy, AUC, Specificity, Recall, Precision, F1 and Kappa of the XGBoost-based classification models on testing data. The values range between 0 (poor performance) and 1 (good performance). **B**-**H** The performance of top 2 (**B**), 5 (**C**), 10 (**D**), 20 (**E**), 40 (**F**), 80 (**G**), and 160 (**H**) specie-based models on the external validation cohort. **I** The performance of top 2, 5, 10, 20, 40, 80, and 160 specie-based models on the external validation data. **J** The performance of top 10 specie-based models on the AGPIBD cohort. **K** SHAP value plot of top 10 important species of XGB-IBD10. **L** Correlations between specific microbial species and IBD. The X-axis represents the log10 (*q* value), calculated using MaAsLin2 based on the differential abundance of identified microbial species in MC-IBD cohort. Negative values on the X-axis represent higher abundance in the control group, while positive values indicate higher abundance in IBD. The Y-axis displays only those microbial species with available abundance data in our samples

The constructed classification analyses yielded various performance metrics, such as accuracy, AUC, specificity, recall, precision, F1, and kappa. The model was trained using 70% of the data, and the remaining 30% served as testing samples (Cohen 1960). The top 160 species-based model (mean [SD] accuracy: 0.9261 [0.0101]) outperformed the top 2 (mean [SD] accuracy: 0.7719 [0.0178]), 5 (mean [SD] accuracy: 0.8157 [0.0135]), 10 (mean [SD] accuracy: 0.8722 [0.0122]), 20 (mean [SD] accuracy: 0.8999 [0.0107]), 40 (mean [SD] accuracy: 0.9174 [0.0053]), and 80 (mean [SD] accuracy: 0.9248 [0.0109]) species-based models on the testing data using tenfold cross-validation (Fig. 3A, Fig. S1, and Table 1). Compared with the top 2 and 5 species-based models, the top 10 species-based model (XGB-IBD10) demonstrated superior performance (accuracy: 0.8066) by external validation with the MC-IBD cohort we collected (Fig. 3B-D). XGB-IBD10 achieved better performance with fewer parameters than that of the top 20, 40, 80, and 160 species-based models (Fig. 3E-I).

**Table 1:** The performance of each classification model

Classifier model	TP^*^	FP^*^	TN^*^	FN^*^	SUM^*^	Accuracy	Specificity	Recall	Precision	F1
Top 2	92	42	32	15	181	0.6851	0.4324	0.8598	0.6866	0.7635
Top 5	100	41	33	7	181	0.7348	0.4459	0.9346	0.7092	0.8065
Top 10 (XGB-IBD10)	101	29	45	6	181	0.8066	0.6081	0.9439	0.7769	0.8523
Top 20	99	35	39	8	181	0.7624	0.5270	0.9252	0.7388	0.8216
Top 40	102	37	37	5	181	0.7680	0.5000	0.9533	0.7338	0.8293
Top 80	101	36	45	6	188	0.7766	0.5556	0.9439	0.7372	0.8279
Top 160	100	36	38	7	181	0.7624	0.5135	0.9346	0.7353	0.8230
Random Forest	91	43	31	16	181	0.6740	0.4189	0.8505	0.6791	0.7552
Decision Tree	93	51	23	14	181	0.6409	0.3108	0.8692	0.6458	0.7410
K Nearest Neighbour	97	51	23	10	181	0.6630	0.3108	0.9065	0.6554	0.7608
Logistic Regression	95	35	39	12	181	0.7403	0.5270	0.8879	0.7308	0.8017
SVM	104	49	25	3	181	0.7127	0.3378	0.9720	0.6797	0.8000
Naives Bayes	93	29	45	14	181	0.7624	0.6081	0.8692	0.7623	0.8122
AE	105	45	29	2	181	0.7403	0.3919	0.9813	0.7000	0.8171
DAE	103	55	19	4	181	0.6740	0.2568	0.9626	0.6519	0.7774
SVAE	93	36	38	14	181	0.7238	0.5135	0.8692	0.7209	0.7881
CNN	90	19	55	17	181	0.8011	0.7432	0.8411	0.8257	0.8333
HMP2-IBD100	105	44	30	2	181	0.7459	0.4054	0.9813	0.7047	0.8203
UMCG IBD-IBD175	98	42	32	9	181	0.7182	0.4324	0.9159	0.7000	0.7935
PRISM-IBD100	100	42	32	7	181	0.7293	0.4324	0.9346	0.7042	0.8032
RISK-IBD21	99	68	6	8	181	0.5801	0.0811	0.9252	0.5928	0.7226
China^#^	86	23	51	21	181	0.7569	0.6892	0.8037	0.7890	0.7963
Other^#^	102	38	36	5	181	0.7624	0.4865	0.9533	0.7286	0.8259

*TP true positive, FP false positive, TN true negative, FN false negative, SUM TP + FP + TN + FN

# The categories “China” and “Other” respectively represent models built based on the Chinese cohort (117 samples from He et al. (2017)) and cohorts from other regions (6,680 samples sourced outside of China). The rest of the models were constructed based on all cohorts

To evaluate the applicability and accuracy of our model to other IBD datasets, we utilized other external validation microbial taxonomic abundance data (AGPIBD; American Gut Project IBS cohort) of IBD (*n* = 220) and healthy individuals (*n* = 101) in the 16S data type obtained from the AGP (American Gut Project; http://americangut.org) (McDonald et al. 2018). Each sample was required to have abundance information for at least one microbial. The model's prediction accuracy was 0.7040 (Fig. 3J).

The SHAP analysis of feature importance in XGB-IBD10 revealed that several species, including *Dorea formicigenerans*, *Oscilibacter massiliensis*, *A. rubiinfantis*, *Fusicatenibacter saccharivorans, Collinsella aerofaciens, Ruminococcus bromii, A. muciniphila*, and *Alistipes finegoldii*, had higher weights in the control group (Fig. 3K and Table S2) than those in the IBD group. Conversely, *Clostridioides difficile* and *Eubacterium oxidoreducens* exhibited higher weights in the IBD group than that in the control group, which had a greater positive impact on predicting the presence of IBD than that of the other features. The higher SHAP values associated with certain features in the IBD group suggest that they are key drivers in the decision-making process of the model for predicting IBD. Furthermore, we used the analytical tool MaAsLin to determine correlations between clinical metadata and microbial community abundance after adjusting the potential confounders in the metagenomic data from MC-IBD cohort and found that *D. formicigenerans* (*q* value = 0.0016), *F. saccharivorans* (*q* value = 2.4 × 10^–9^), *C. aerofaciens* (*q* value = 0.0425), *R. bromii* (*q* value = 3.5 × 10^–10^), and *A. finegoldii* (*q* value = 1.6 × 10^–5^) had higher abundances in the control group than those in the IBD group (Fig. 3L). However, no significant difference was observed in the *A. muciniphila* and *C. difficile* abundances. Limited information was available on *O. massiliensis*, *A. rubiinfantis,* and *E. oxidoreducens*. Consistent with a previous report (Pascal et al. 2017), the decreased *C. aerofaciens* abundance in IBD was correlated with decreased *Collinsella* levels associated with IBD (Fig. 3K). Additionally, *A. muciniphila* has been recognised as a potential probiotic, and modified versions of *A. muciniphila* have exhibited therapeutic effects in intestinal diseases when exposed to an external magnetic field (Wang et al. 2023; Zhang et al. 2021). The decrease in *R. bromii* in patients with IBD was consistent with the low abundance of *R. gnavus* observed in patients with IBD (Fig. 3K) (Gevers et al. 2014; Hall et al. 2017). *O. massiliensis* and *A. rubiinfantis* were newly identified IBD biomarkers in our model and exhibited lower expression in IBD samples than that in the control samples.

### XGB-IBD10 achieves a better performance than the model solely based on metagenomic or 16S sequencing data

**Fig. 4:**
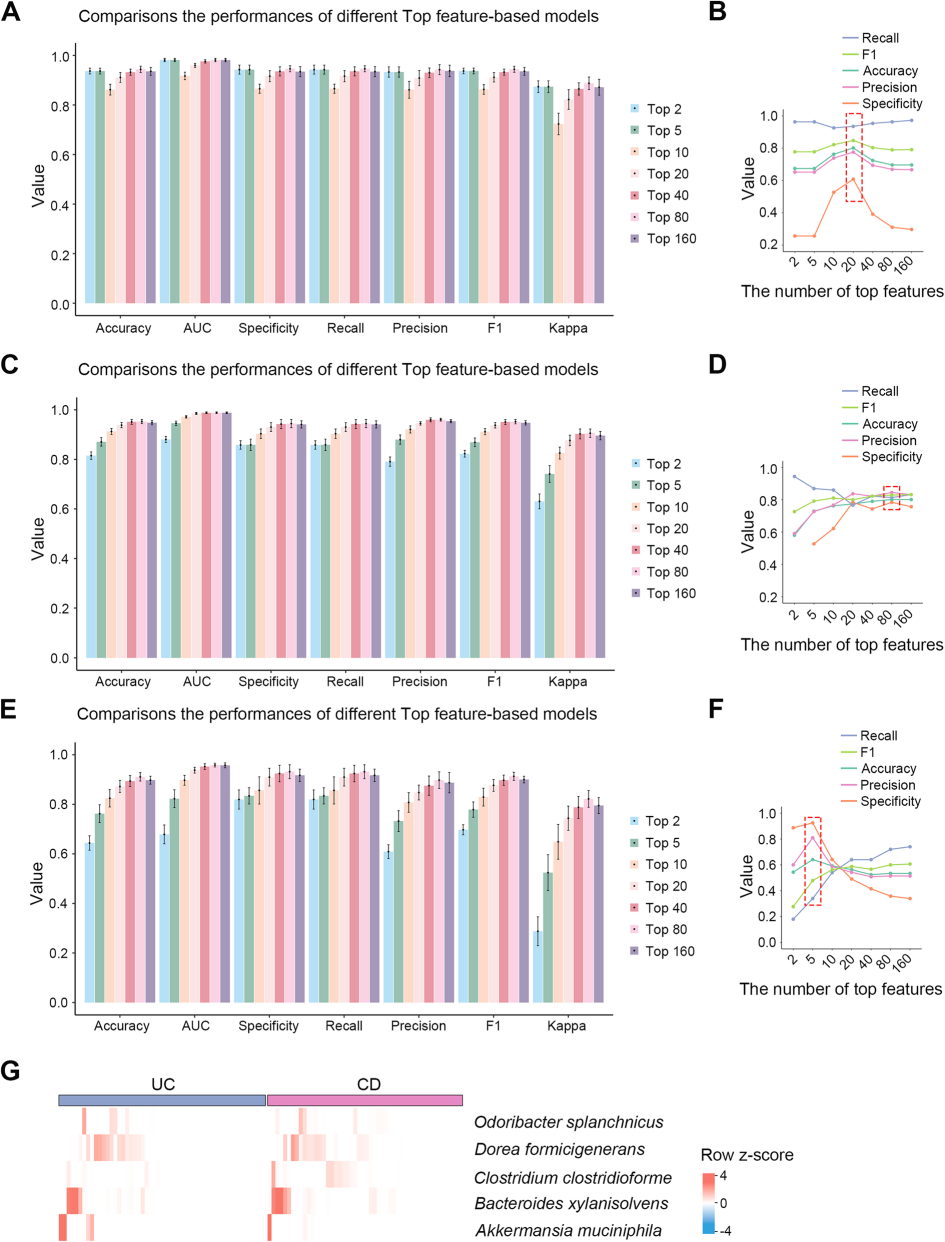
XGB-IBD10 achieves a better performance than the metagenomic or 16S sequencing data-based model. **A** The histogram comparisons of the Accuracy, AUC, Specificity, Recall, Precision, F1 and Kappa of the metagenomic data-based classification models on testing data. The values range between 0 (poor performance) and 1 (good performance). **B** The performance of top 2, 5, 10, 20, 40, 80, and 160 specie- metagenomic data-based models on the external validation data. **C** The histogram comparisons of the Accuracy, AUC, Specificity, Recall, Precision, F1 and Kappa of the 16S data-based models on the testing data. The values range between 0 (poor performance) and 1 (good performance). **D** The performance of top 2, 5, 10, 20, 40, 80, and 160 specie- 16S data-based models on the external validation data. The top 2 specie- 16S data-based models has a specificity of 0.054, which is why it is not displayed in the figure. **E** The histogram comparisons of the Accuracy, AUC, Specificity, Recall, Precision, F1 and Kappa of the UC and CD data -based classification models on the testing data. The values range between 0 (poor performance) and 1 (good performance). **F** The performance of top 2, 5, 10, 20, 40, 80, and 160 species- UC and CD data-based models on the external validation data (only utilize the data of 103 IBD patients). **G** The top 5 microbial signature abundance patterns in the external validation data. Row Z-score heatmaps were generated using microbial abundance values in the external validation

Most previously reported models have been established using metagenomic or 16S sequencing data (Sen et al. 2024; Seo et al. 2023). We explored whether the predictive performance could be obtained by combining metagenomic and 16S rRNA sequencing data in our model. For the metagenomic data, the model constructed using the top 80 features had the highest accuracy of 0.9437 [SD 0.0168] in the testing data (Fig. 4A) but achieved an accuracy of 0.6961 in the MC-IBD cohort (with a top 20 feature accuracy of 0.8011) (Fig. 4B). For the 16S sequencing data, the model constructed using the top 80 features had the highest accuracy of 0.9528 [SD = 0.0113] in the testing dataset (Fig. 4C) but had an accuracy of 0.8011 in the MC-IBD cohort (Fig. 4D). Therefore, XGB-IBD10, which is based on combined metagenomic and 16S sequencing data, outperformed the models that relied solely on metagenomic or 16S sequencing.

### XGBoost exhibits moderate performance in classifying UC and CD

Distinct microbiome profiles exist in individuals with UC and CD (Morgan et al. 2012; Pascal et al. 2017). To assess whether XGBoost can be used to construct a model to efficiently distinguish UC from CD, we built classification models using a dataset comprising 1,858 IBD samples (UC: 485; CD: 1373) from Integrative Human Microbiome Project (HMP2) and NIHMS1510763, among which 70% of the data were used for training and the remaining 30% were used for testing. The top 250 microbial species with the highest variation in abundance were selected from HMP2, NIHMS1510763, SRP057027, and He et al. (2017) metagenomic cohorts and calculated the SHAP values for the model (Table S3). Our results revealed that the model incorporating the top 80 features exhibited the highest accuracy of 0.9110 [SD = 0.0225] on the testing data (Fig. 4E). However, the model incorporating the top five microbial species exhibited the highest accuracy (0.6408) in the MC-IBD cohort (Fig. 4F and Table S4). The prediction accuracy of our model was lower for the subtype classifications of UC and CD than that for the overall IBD classification. Fig. 3 and Fig. 4 were constructed under fundamentally different data conditions, necessitating distinct top feature. Fig. 3 evaluates the XGB-IBD10 model trained on metagenomic and 16S sequencing data. Fig. 4 intentionally tests single-type datasets (e.g., metagenomic-only in Fig. 4 A-B, 16S-only in Fig. 4 C-D, UC/CD-only in Fig. 4 E-F) to simulate real-world scenarios with limited data types. Here, no fixed feature set (including XGB-IBD10) consistently outperformed others because: (i) the model solely based on metagenomic or 16S sequencing data lack species-resolution; (ii) UC/CD-specific models prioritize subtype-discriminative features. At last, the XGB-IBD10 model achieves optimal overall performance with a minimal number of features. In the abundance analysis of the external 103 IBD validation datasets, we observed that the microbial species *D. formicigenerans* and *A. muciniphila* were more abundant in individuals with UC, whereas *Odoribacter splanchnicus*, *Clostridium clostridioforme*, and *Bacteroides xylanisolvens* were more abundant in individuals with CD (Fig. 4G and Table S4).

### XGB-IBD10 achieves a better performance than that of other machine learning and deep learning-based methods

**Fig. 5:**
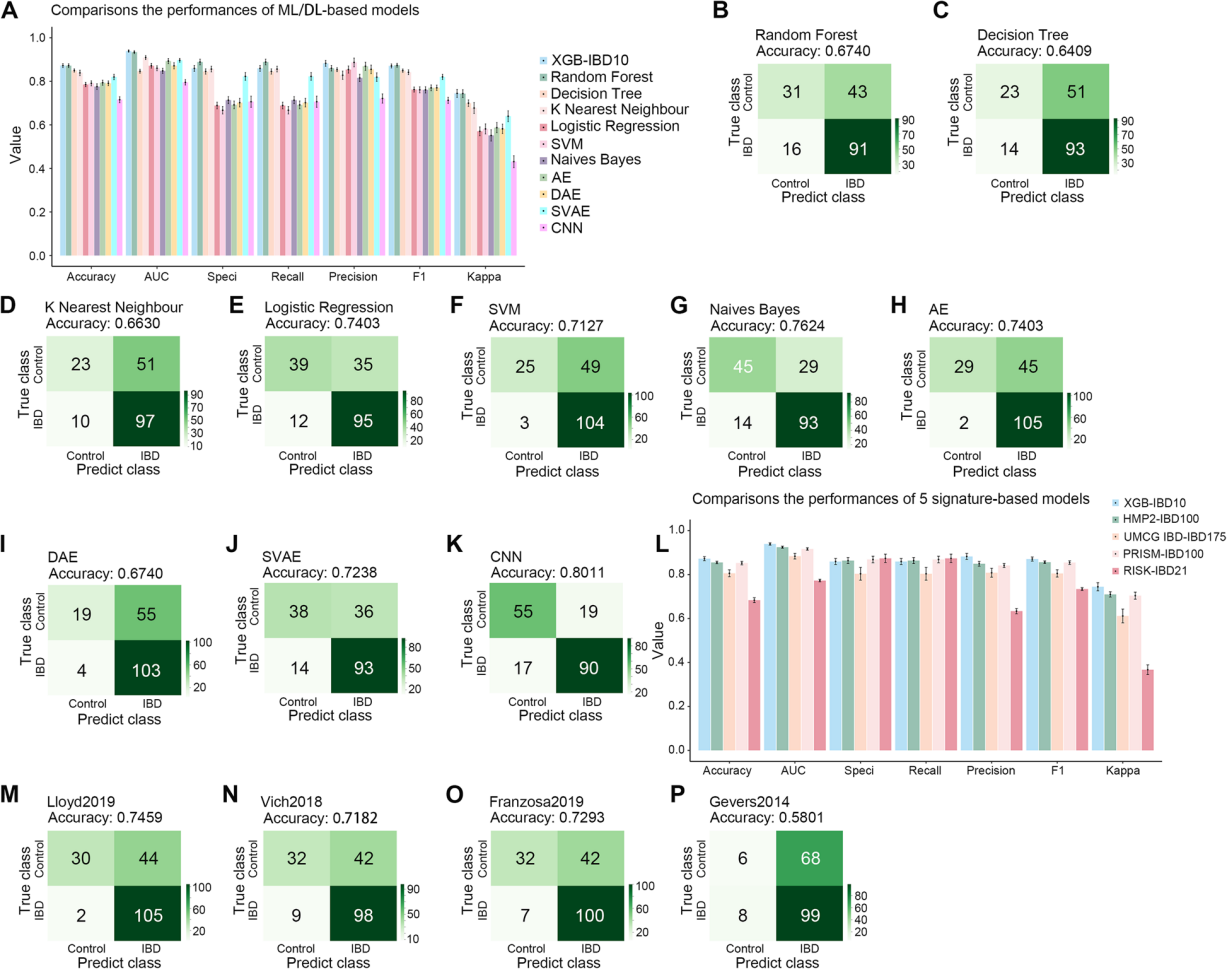
XGB-IBD10 achieves a better performance than other algorithms or species signature-based models. **A** The histogram comparisons of the Accuracy, AUC, Specificity, Recall, Precision, F1 and Kappa of the machine learning and deep learning-based classification models on testing data, those values range between 0 (poor performance) and 1 (good performance). **B**-**K** Comparison of the performance of XGB-IBD10 with Random Forest (**B**), Decision Tree (**C**), K nearest Neighbour (**D**), Logistic Regression (**E**), SVM (**F**), Naives Bayes (**G**), AE (**H**), DAE (**I**), SVAE (**J**), and CNN (**K**) algorithm-based models on the external validation data. **L** The histogram comparisons of the Accuracy, AUC, Specificity, Recall, Precision, F1 and Kappa of the other species signature-based classification models on testing data. The values range between 0 (poor performance) and 1 (good performance). **M-P** Comparison of the performance of XGB-IBD10 with HMP2-IBD100 (**M**), UMCG IBD-IBD175 (**N**), PRISM-IBD100 (**O**), and RISK-IBD21 (**P**) Algorithm-based models on the external validation cohort. Confusion matrix detailing the true positive (right lower), true negative (left upper), false positive (right upper), and false negative (left lower) predictions from XGB-IBD10. Accuracy = (true positive + true negative)/total

To ensure high accuracy, we compared the performance of our model with that of various machine-learning methods. Among these methods, random forest performed well in predicting disease relapse (Sarrabayrouse et al. 2021). Our XGB-IBD10 model (mean [SD] accuracy: 0.8722 [0.0122]) performance was comparable with that of random forest (mean [SD] accuracy: 0.8718 [0.0119]) on the testing data, outperforming other methods such as decision tree, k-nearest neighbor, logistic regression, support vector machines (SVM), and Naïve Bayes (Fig. 5A). However, XGB-IBD10 (accuracy: 0.8066) achieved superior performance on the MC-IBD cohort (Fig. 5B-G and Table 1). Although the random forest model performed best on the testing data, it had lower accuracy in the MC-IBD cohort, indicating possible overfitting. Contrastingly, the logistic regression and Naïve Bayes models exhibited good performance in the MC-IBD cohort.

Considering the potential variations in species abundance within the microbial datasets, we utilized deep learning algorithms to transform the data. We then employed the transformed data to construct the models and evaluated their performance. We explored multiple deep learning methods, such as autoencoder (AE), denoising autoencoder (DAE), stacked variational autoencoder (SVAE), and convolutional neural network (CNN), to enhance the performance of our model. For the model construction, we selected the multilayer perceptron (MLP) algorithm, which is a type of feedforward artificial neural network with one hidden layer. Deep learning uses the abundance information of all samples as input data and provides the transformed intermediate data to the MLP-based model for model building and accuracy prediction. XGB-IBD10 had better performance compared to AE (mean [SD] accuracy: 0.7936 [0.0155]), DAE (mean [SD] accuracy: 0.7909 [0.0143]), SVAE (mean [SD] accuracy: 0.8199 [0.0165]), and CNN (mean [SD] accuracy: 0.7155 [0.0182]) on the testing data (Fig. 5A). Furthermore, when compared to the AE (accuracy: 0.7403), DAE (accuracy: 0.6740), SVAE (accuracy: 0.7238), and CNN (accuracy: 0.8011) based models, XGB-IBD10 achieved similar or better performance in the MC-IBD cohort (Fig. 5H-K and Table 1). The MLP-based classification model performance was weakened when the microbial data-based classification model was integrated with multiple deep learning methods. Among the deep learning methods, SVAE, a stacked VAE model with Gaussian hidden layers, achieved better performance than that of the other models in MC-IBD cohort. Collectively, XGB-IBD10 exhibited good performance for the testing and MC-IBD cohorts.

### XGB-IBD10 has a better performance than that of other species signature-based methods

To construct a machine learning model with high accuracy and efficiency for the MC-IBD cohort, we utilized the XGBoost algorithm and incorporated nine distinct cohorts considering microbial species from published metagenomic analyses. The XGB-IBD10 model (mean [SD] accuracy: 0.8722 [0.0122]) outperformed other models based on species signatures on the testing data, including the HMP2-IBD100 (mean [SD] accuracy: 0.8549 [0.0079]), UMCG IBD-IBD175 (mean [SD] accuracy: 0.8058 [0.0208]), PRISM-IBD100 (mean [SD] accuracy: 0.8521 [0.0106]), and RISK-IBD21 (mean [SD] accuracy: 0.6836 [0.0147]) (Fig. 5L). Moreover, compared with the models based on HMP2-IBD100 (accuracy: 0.7459), UMCG IBD-IBD175 (accuracy: 0.7182), PRISM-IBD100 (accuracy: 0.7293), and RISK-IBD21 (accuracy: 0.5801), XGB-IBD10 (accuracy: 0.8066) demonstrated better performance on our external 181 IBD samples (Fig. 5M-P and Table 1).

### Superior performance of the whole cohort-based classification model

**Fig. 6:**
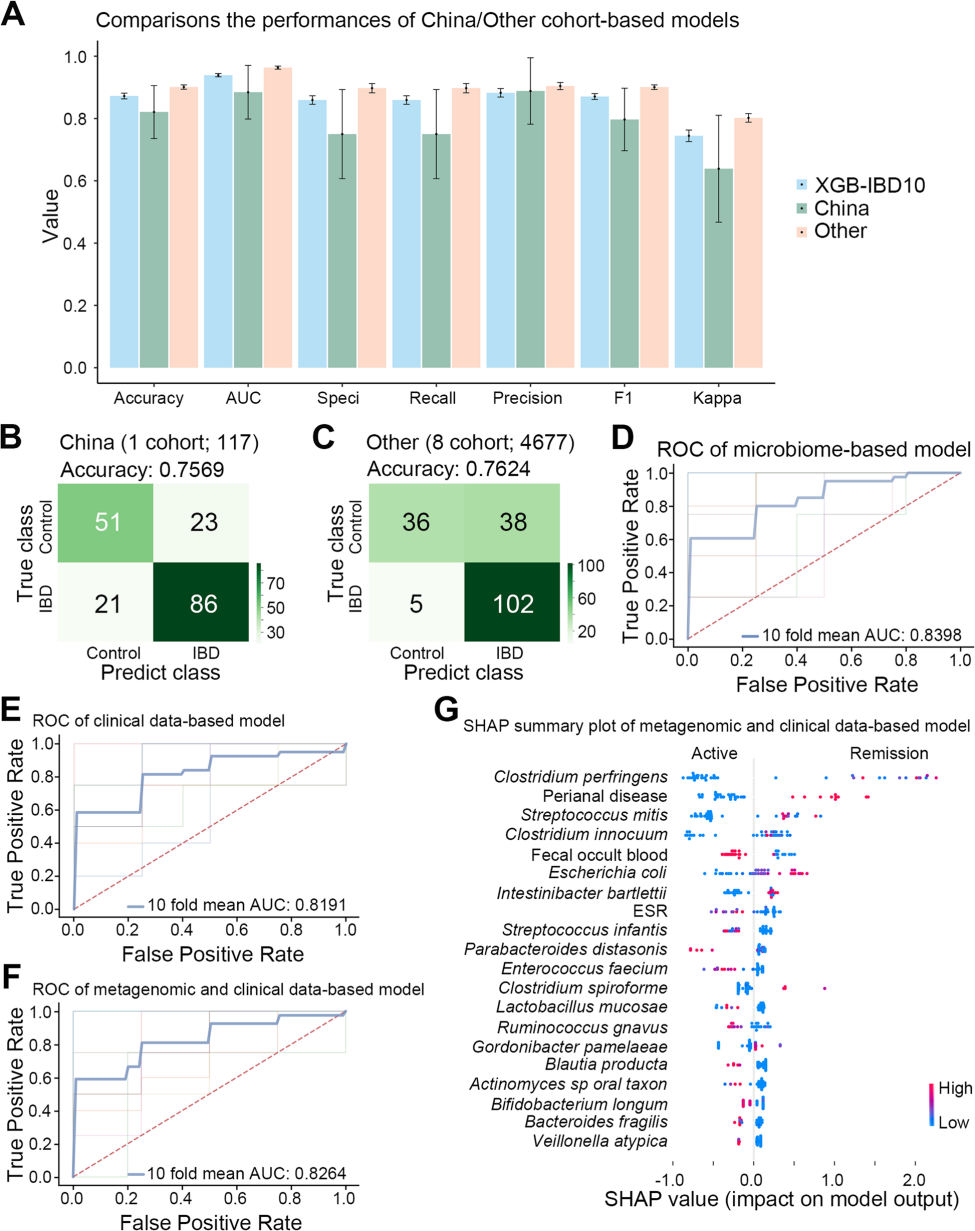
The microbiome-based model outperforms clinical data-based models. **A** The histogram comparisons of the Accuracy, AUC, Specificity, Recall, Precision, F1 and Kappa of the different geographic-based classification models on testing data, those values range between 0 (poor performance) and 1 (good performance). **B** and **C** XGB-IBD10 (all cohort-based models) performed better than Chinese (**B**) and other (**C**) cohort-based models on the external validation data. **D** Microbiome-based ROC curves for classification of active and remission individuals. **E** Clinical data-based ROC curves for classification of active and remission individuals. **F** Metagenomic and clinical data-based ROC curves for classification of active and remission individuals. **G** SHAP value plot of top 20 important species of metagenomic and clinical data-based model. Confusion matrix detailing the true positive (right lower), true negative (left upper), false positive (right upper), and false negative (left lower) predictions from XGB-IBD10. Accuracy = (true positive + true negative)/total

By considering the variations in microbial species across different geographic regions, we included cohorts from distinct regions to enhance the performance of our model (Metwaly et al. 2022). The overall cohort-based model (XGB-IBD10) demonstrated superior performance (mean accuracy: 0.8722 [SD: 0.0122]) compared with that of the Chinese cohort-based models (mean accuracy: 0.8208 [SD: 0.1126]) (Fig. 6A). Additionally, XGB-IBD10 achieved good performance (accuracy: 0.8066) on our MC-IBD 181 samples, outperforming the Chinese (accuracy: 0.7569) and other cohorts (accuracy: 0.7624) (Fig. 6B-C, and Table 1). Furthermore, the entire cohort-based classification model outperformed the partial cohort-based methods. The model achieved comparable accuracy with a larger sample size (*n* = 4,677) from other regions and with a smaller sample size (*n* = 117) from the Chinese cohort, suggesting that there is potential to significantly enhance the predictive performance of the model by obtaining high-quality data from the Chinese population.

### Metagenomic data facilitates the classification of IBD active and remission states

The detection of active IBD primarily relies on endoscopy-based monitoring because biomarkers alone are insufficient (Singh et al. 2023). In this study, we aimed to construct separate classification models using clinical and metagenomic data to distinguish between active and remission states of IBD. We incorporated biological markers, such as CRP, erythrocyte sedimentation rate, and fecal occult blood, to develop an active IBD classification model (Table 2, Table S5). A significant difference was found in sex distribution (*p* = 0.040), whereas no significant variations were observed in age composition (*p* = 0.17). There were significant differences in perianal disease (*p* = 0.0038), complications (*p* = 0.032), CRP levels (*p* < 0.001), erythrocyte sedimentation rates (*p* = 0.007), and fecal occult blood levels (*p* < 0.001) (Table 3, Table S5). The classification model using only metagenomic data demonstrated better performance (mean AUC: 0.8398) in distinguishing between active and remission states than that of models using clinical data (mean AUC: 0.8191) or a combination of both (mean AUC: 0.8264) (Fig. 6D, F). We observed a significant correlation between active IBD and higher levels of *Parabacteroides distasonis*, *Enterococcus faecium*, fecal occult blood, CRP, and erythrocyte sedimentation rate (Fig. 6G, S2, S3 A-B, Table S6). However, age and fecal calprotectin had limited discriminatory power in the model classification (Fig. S3C-D, Table S5). However, individuals who had undergone surgery for anal fistulas were more likely to be classified as in remission, possibly indicating increased awareness and early treatment seeking when complications arose (Fig. 6G, S3E, Table S5). Variances in fecal occult blood, complications, and upper gastrointestinal involvement also influenced the classification of IBD states (Fig. S3 F,H, Table S5). Additionally, among patients with IBD, females were more likely to be classified as being in the active state (*p* = 0.04) (Fig. S3I and Table S5). By incorporating metagenomic data, the microbiome-based model improved the mean AUC for predicting IBD states compared to models relying solely on clinical data, highlighting the value of the XGBoost-based approach for integrating various IBD-related biomarkers and enhancing the prediction accuracy.

**Table 2:** Characteristics of 103 IBD patients

	UC (N = 53)	CD (N = 50)	*P*-value
Behavior			
Chronic relapsing	40 (75.5%)	0 (0%)	< 0.001
Initial onset	9 (17.0%)	0 (0%)	
Non-stricturing non-penetrating	2 (3.8%)	20 (40.0%)	
Stricture	1 (1.9%)	21 (42.0%)	
Penetrating	0 (0%)	8 (16.0%)	
Without	1 (1.9%)	1 (2.0%)	
Location			
Colon	1 (1.9%)	8 (16.0%)	< 0.001
Extensive colitis	38 (71.7%)	0 (0%)	
Ileocolon	2 (3.8%)	31 (62.0%)	
Left-sided colitis	8 (15.1%)	0 (0%)	
Rectum	3 (5.7%)	0 (0%)	
Ileum	0 (0%)	11 (22.0%)	
without	1 (1.9%)	0 (0%)	
Mayo score^#^			
Mean (SD)	6.15 (3.74)	No data	< 0.001
Median [Min, Max]	6.00 [0, 12.0]	No data	
CDAI^#^			
Mean (SD)	No data	105 (93.0)	< 0.001
Median [Min, Max]	No data	67.5 [0, 367]	
Type			
Active	43 (81.1%)	15 (30.0%)	< 0.001
Remission	10 (18.9%)	35 (70.0%)	
Age			
Mean (SD)	43.9 (15.2)	32.7 (13.3)	< 0.001
Median [Min, Max]	40.0 [17.0, 80.0]	28.5 [18.0, 65.0]	
Gender			
Male	34 (64.2%)	36 (72.0%)	0.521
Female	19 (35.8%)	14 (28.0%)	
Upper gastrointestial involvement			
With	1 (1.9%)	9 (18.0%)	0.01
Without	50 (94.3%)	41 (82.0%)	
No data	2 (3.8%)	0 (0%)	
Perianal disease			
With	5 (9.4%)	27 (54.0%)	< 0.001
Without	46 (86.8%)	23 (46.0%)	
No data	2 (3.8%)	0 (0%)	
Complications			
Carcinogenesis	5 (9.4%)	1 (2.0%)	0.00219
Extraintestinal manifestations	3 (5.7%)	1 (2.0%)	
Stricture	2 (3.8%)	10 (20.0%)	
Surgery	2 (3.8%)	5 (10.0%)	
Penetrating	0 (0%)	6 (12.0%)	
without	41 (77.4%)	26 (52.0%)	
No data	0 (0%)	1 (2.0%)	
C-reactive protein			
Mean (SD)	1.25 (1.93)	1.99 (3.30)	0.174
Median [Min, Max]	0.440 [0, 8.33]	0.360 [0, 13.8]	
Erythrocyte sedimentation rate			
Mean (SD)	16.0 (16.8)	15.9 (20.2)	0.987
Median [Min, Max]	13.0 [0, 73.0]	7.50 [0, 89.0]	
Fecal calprotectin			
Mean (SD)	36.9 (21.2)	42.3 (19.6)	0.186
Median [Min, Max]	37.5 [0, 60.0]	37.5 [0, 60.0]	
Fecal occult blood			
Positive	40 (75.5%)	23 (46.0%)	0.00133
Negative	11 (20.8%)	27 (54.0%)	
No data	2 (3.8%)	0 (0%)	

^#^CDAI (Crohn's Disease Activity Index): This is a research tool used to quantify the symptoms and features of Crohn's Disease. It helps in assessing disease severity and is commonly used in clinical trials to determine the efficacy of treatments

^#^Mayo Score: This is a scoring system used to assess the severity of ulcerative colitis. It includes subscores for stool frequency, rectal bleeding, endoscopic findings, and physician's global assessment, providing a comprehensive overview of disease activity

**Table 3:** Characteristics of 107 IBD patients

	Patients with active IBD (*N* = 60)	IBD patients in remission (*N* = 47)	*P* value *
UC, n (%)	43 (71.7)	10 (21.3)	< 0.001
-Mayo score	7.61 (3~12)	2.00 (1~3)	< 0.001
-No data, n (%)	2 (3.3)	3 (6.4)	0.015
CD, n (%)	15 (25.0)	35 (74.5)	< 0.001
-CDAI	223.60 (70~367)	55.34 (0~149)	< 0.001
-No data, n (%)	0 (0)	1 (2.1)	0.54
Male, n (%)	36 (60.0)	37 (78.7)	0.040
Age (median and range)	40.42 (17~80)	35.4 (18~66)	0.17
Upper gastrointestinal involvement, n (%)	2 (3.3)	9(19.1)	0.065
Perianal disease, n (%)	11 (18.3)	22 (46.8)	0.0038
Complications, n (%)	16 (26.7)	22 (46.8)	0.032
-stricture, n (%)	4 (6.7)	10 (21.3)	0.17
-Extraintestinal manifestations, n (%)	2 (3.3)	2 (4.3)	0.81
-penetrating, n (%)	2 (3.3)	4 (8.5)	0.60
-surgery, n (%)	3 (5)	5 (10.6)	0.72
-carcinogenesis, n (%)	5 (8.3)	1 (2.1)	0.037
C-reactive protein (median and range)	2.19 (0~13.8)	0.87 (0.1~9.22)	< 0.001
Erythrocyte sedimentation rate (median and range)	20.5 (0~89)	9.94 (1~59)	0.007
Fecal calprotectin (≥ 60), n (%)	24 (40.0)	19 (40.4)	0.97
Fecal occult blood, n (%)	46 (76.7)	20 (42.6)	< 0.001

^*^We used Mann–whitney tests for differences between IBD patients in active and remission

### XGBoost exhibits good performance in distinguishing IBD and IBS

**Fig. 7:**
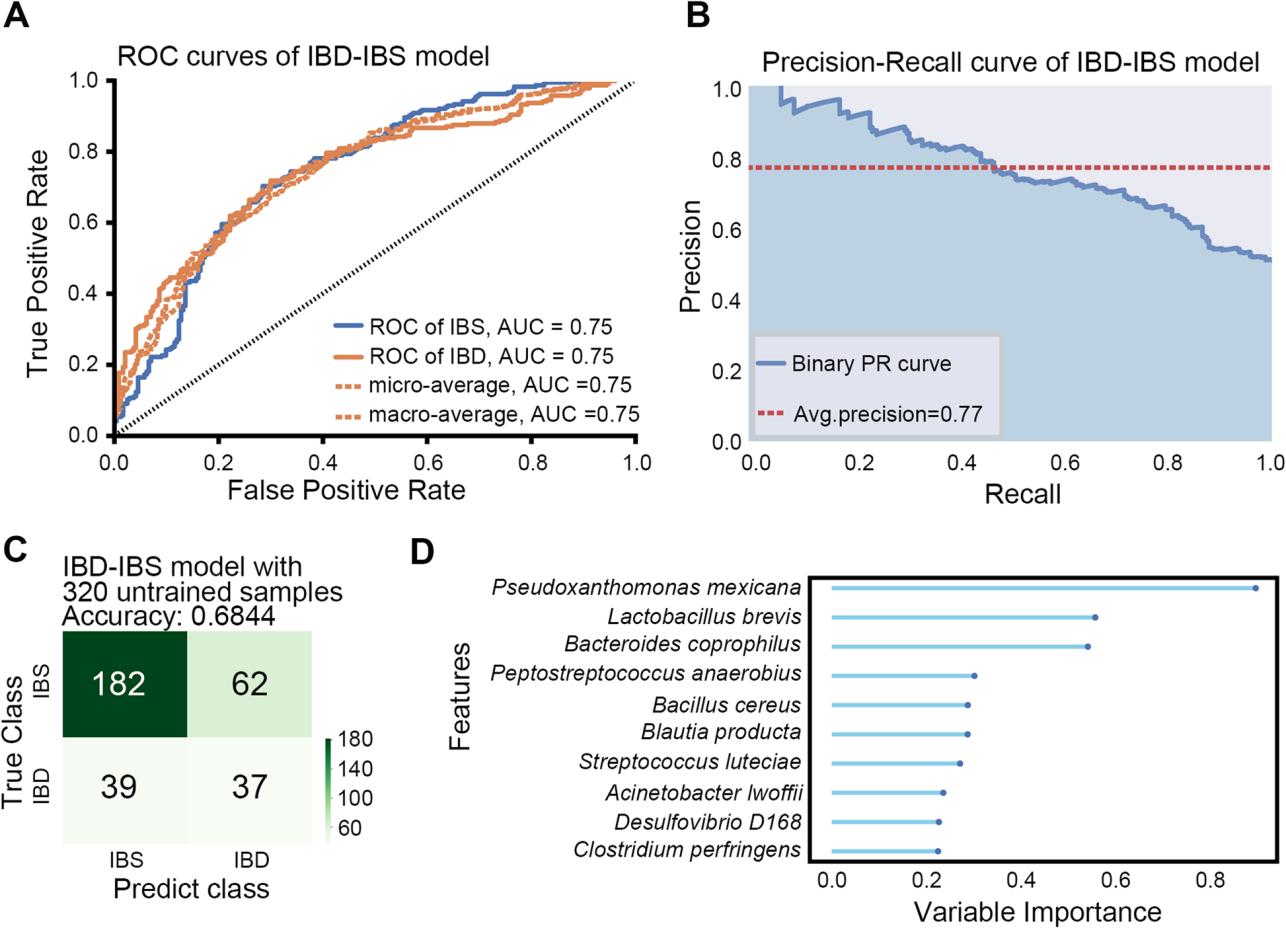
XGBoost exhibits good performance in classifying IBD and IBS. **A** XGBoost-based ROC curves for classification of IBD and IBS individuals. **B** XGBoost-based Precision-Recall curves for classification of IBD and IBS individuals. **C** XGBoost-based model performed good on the untrained data. **D** The variable importance of top 10 important species of XGBoost-based IBD-IBS model

It has been reported that the intestinal microbiota in irritable bowel syndrome (IBS) patients are distinct from those in IBD patients (Rengarajan et al. 2020). Then, we attempted to build a model to distinguish between IBD and IBS. The microbial taxonomic abundance data from the AGPIBDIBS cohort (part of the American Gut Project) were utilized to construct an XGBoost-based classification model. This dataset included samples from 255 IBD patients and 811 individuals with IBS, derived from 16S rRNA sequencing (doi: 10.1128/mSystems.00031-18). As mentioned in methods, we used 70% of the data for training and validation, with the abundance data of all microbial used for model construction. The remaining 30% of the data, which was not used for model training, was used for testing. The resulting receiver operating characteristic (ROC) curve had an AUC of 0.75, and the Precision-Recall curve showed an average precision of 0.77 (Fig. 7A-B). The accuracy in the untrained 30% samples reached 0.6844 (Fig. 7C). The most important microbial in the model's classification process were *Pseudoxanthomonas mexicana*, *Lactobacillus brevis*, and *Bacteroides coprophilus* (Fig. 7D). *Lactobacillus* were found to enhance intestinal barrier integrity and reduce gastrointestinal infections and IBD phenotypes (Hou et al. 2018). *Bacteroides* were found to be increased in IBS patients, especially those with IBS diarrhea (Pittayanon et al. 2019). These results suggest that our model can nicely distinguish IBD from IBS.

## Discussion

Distinct microbial species are associated with IBD compared with those causing other gastrointestinal illnesses (Vich Vila et al. 2018). However, most IBD studies have not analyzed the microbial species at the strain level because of technical limitations (Pittayanon et al. 2020). In this study, we obtained microbiome profiles at the species level using metagenomic sequencing and identified a 10-species signature (XGB-IBD10) for IBD diagnosis. XGB-IBD10 outperformed other models (RISK-IBD21, UMCG IBD-IBD175, HMP2-IBD100, and PRISM-IBD100), in terms of accuracy, AUC, specificity, sensitivity, recall, precision, F1, and kappa. Blind testing demonstrated that XGB-IBD10 achieved higher accuracy (0.8066) in our MC-IBD cohort than that of the control group.

Among the 10 XGB-IBD10 species, *C. aerofaciens* and *A. muciniphila* have been associated with healthy mucosae (Zhang et al. 2021). Notably, *A. muciniphila* has garnered clinical interest for its probiotic potential and demonstrated capacity to accelerate intestinal and liver regeneration (Hu et al. 2025; Kang et al. 2024). Higher *C. aerofaciens* and *A. muciniphila* abundance has been reported in active rheumatoid arthritis patients (Chiang et al. 2019). Age-related differences may contribute to the variations in the abundance of these species, although confirmation is hindered by the limited availability of age information. The functional correlation between these biomarkers and IBD pathogenesis remains unclear and requires further investigation.

Variation in microbial species in IBD is influenced by various factors, including geography, disease activity status, and environmental (Mayorga et al. 2022; Wang et al. 2022). Despite recognizing the differences in bacterial abundance between patients with IBD and controls, inconsistent results and methodologies preclude definitive conclusions (Pittayanon et al. 2020). Therefore, we built a model using data from patients at various locations to capture a wide range of microbial diversity and environmental factors associated with IBD. By combining data from different regions, our model aimed to identify consistent microbial signatures associated with IBD across populations for better generalizability and reliability. External validation was performed using samples from a single hospital representing a population with a relatively uniform geographic distribution. Using an external validation set from a similar geographic area, we minimized the potential confounding effects of regional environmental factors on the microbiome. Most studies focused on populations in Western countries. Notably, cases of IBD in Chile and Spain have confirmed the reduced abundance of *F. prausnitzii* and increased *E. coli* in the diagnosis of IBD (Chamorro et al. 2021; Vidal et al. 2015). The 117 Chinese samples achieved a prediction effect comparable to that of models constructed using 4,677 samples from other regions, suggesting the potential for further improvement by including additional cohorts. However, the potential bias introduced by choosing features based on abundance variance in the public cohorts may result in the selection of species that are strong predictors of IBD due to their correlation with the cohorts, highlighting a limitation of our feature selection method. We improved the disease classification accuracy by categorizing patients with IBD into active and remission states using clinical and metagenomic data. Elevated CRP levels and fecal occult blood are commonly associated with active IBD (Singh et al. 2023). XGBoost-based model also exhibits good performance in classifying IBD and IBS.

Previous studies has used microbial species to discriminate UC from CD (Morgan et al. 2012; Pascal et al. 2017). A Random Forest classification model was developed using metagenomic data for the control, UC, and CD classifications (Franzosa et al. 2019). These models had 65.2% and 47.7% accuracy in fivefold cross-validation and independent validation, respectively (Franzosa et al. 2019). Bacterial biomarkers have been used for distinguishing UC from CD with 77% accuracy (Chamorro et al. 2021). Our model aimed to distinguish patients with UC and those with CD, regardless of dysbiosis, to aid the initial diagnosis of the IBD subcategories. Our classification model for UC and CD demonstrated an accuracy of 0.9110 [SD 0.0225] for the test data. Furthermore, it achieved the highest accuracy of 0.6408 with the top five features in the external validation dataset. Overall, our UC and CD classification models outperformed the previous models.

Notably, *A. muciniphila* and *C. aerofaciens*, two species associated with health in the XGB-IBD10 context, may have dual roles in the pathogenesis of inflammatory bowel disease (IBD). A diacyl phospholipid (a15:0-i15:0 PE) derived from *A. muciniphila*, which activates the TLR2-TLR1 pathway, is responsible for its immunomodulatory effects by selectively inducing cytokine production and modulating immune activation thresholds (Bae et al. 2022). The unique low-dose signaling of this lipid through a non-canonical TLR heterodimer elucidates *A. muciniphila*'s capacity to regulate immunological tone, thereby linking its membrane components to immune responses specific to the disease. *A. muciniphila* facilitates the degradation of mucins into acetate, thereby enhancing gut barrier function (Trastoy et al. 2020). Additionally, *A. muciniphila* inhibits tryptophan metabolism via the AhR/β-catenin signaling pathway, contributing to the suppression of colorectal cancer progression (Zhang et al. 2023). Similarly, *C. aerofaciens* produces propionate, a short-chain fatty acid with anti-inflammatory properties. The butanol extract from anaerobic monocultures of *C. aerofaciens* ATCC 25986 exhibited significant pro-inflammatory activity by inducing TNFα (Kwon et al. 2023).

Translating the XGB-IBD10 model into clinical practice requires addressing two challenges: 1. Generalizability across ethnicities and mechanistic validation. A hypothetical pipeline could integrate longitudinal stool testing with machine learning-based risk scores to guide preemptive interventions. For instance, patients showing *A. muciniphila* depletion and elevated CRP might receive targeted mucin supplements or next-generation probiotics; 2. Future studies should validate these signatures in interventional cohorts and explore synergies with host genomics/metabolomics.

In summary, effective prediction methods utilizing biomarkers and machine learning are crucial for identifying IBD symptoms. By analyzing a wide range of data points, including clinical metrics and detailed metagenomic information, our model enables a more accurate assessment of a patient’s condition, achieving higher diagnostic accuracy compared to traditional methods that typically rely on fewer criteria. This model will aid researchers in pinpointing key biological and clinical markers associated with IBD, offering valuable insights into the underlying mechanisms of the disease. Such understanding is essential for developing targeted therapies and enhancing disease management strategies.

## Methods

### Patient recruitment and shotgun metagenomic sequencing

First, 181 fecal samples were collected from patients at the Peking University Third Hospital in Beijing, China. Among these samples, 107 and 74 were from individuals with or without IBD, respectively. All patients with IBD were confirmed through endoscopy, whereas the other participants were asymptomatic or had no symptoms of gut disturbance during colonoscopy. The IBD status of the participants with IBD was determined using endoscopy. Inclusion Criteria: (1). Age between 17 and 80 years; (2). IBD disease was diagnosed through endoscopic examination and histopathological confirmation; (3). Classified as having active disease based on the Mayo score for UC or the best Crohn's Disease Activity Index (CDAI) for CD; (4). Patients without a diagnosis of IBD (healthy control group) were confirmed by endoscopic and histopathological examinations. Exclusion Criteria: (1). The major complications include gastrointestinal bleeding, intestinal obstruction, severe infections, and tumors (2). Individuals at high risk of hereditary colorectal cancer, including those with a genetic history of gastrointestinal tumours and familial adenomatous polyposis (3). Fibroblast Activation Protein or Peutz-Jeghers Syndrome; (4). History of gastrointestinal, biliary, or other abdominal surgery; (5). Positive for cardiovascular, endocrine, hematological, or immune system disorders; (6). Undergone invasive medical interventions within the last 3 months (7). Inability to comply with an examination or failure to provide informed consent. Information on complications, upper gastrointestinal involvement, and perianal disease (history of surgical treatment for anal fistulas) was collected from 107 patients with IBD during outpatient visits or endoscopies. Demographic and clinical data included age, sex, and complications related to disease behavior. The clinical status was defined as the Mayo score for patients with UC and the best CDAI score for patients with CD. Laboratory evaluations included CRP levels, erythrocyte sedimentation rate, fecal calprotectin levels, and fecal occult blood. The study was approved by the Ethics Committee of the Peking University Third Hospital (MR-11–22–000625) and samples were collected between March 2021 and May 2023. Written informed consents were obtained from all participants or their guardians before participating in the study.

Stool samples were stored at −80 ℃ after being stored at 4 ℃ for < 24 h until DNA extraction. The microbial sequencing data composition, consisting of MC-IBD cohort, was summarised by age and sex using violin and bar plots. Whole DNA was extracted using the cetyltrimethylammonium bromide method. DNA libraries were prepared using the NEBNext UltraTM DNA Library Prep Kit (New England Biolabs, Ipswich, MA, USA) and sequenced using an Illumina HiSeq platform (Illumina, San Diego, CA, USA), generating approximately 6 G paired-end reads (2 × 150 bp) for each sample.

KneadData (v. 0.10.0) (http://huttenhower.sph.harvard.edu/kneaddata) was used to filter the human genome (hg37dec_v0.1.1 and human_hg38_refMrna) and the ribosomal sequences (SILVA_128_LSUParc_SSUParc_ribosomal_RNA) from the sequencing reads to analyze the microbial sequencing data. MetaPhlAn4 (v. 4.0.2) (https://huttenhower.sph.harvard.edu/metaphlan/) was used to generate taxonomic profiles (database: mpa_v30_CHOCOPhlAn_201901) from shotgun metagenomes (Beghini et al. 2021). To normalize the microbial sequencing data, clade coverages were subsequently standardised across all identified clades to determine the relative abundance of each taxon, as previously described (Beghini et al. 2021). Phyloseq (v. 1.40.0) was used to determine alpha diversity (Abundance-based coverage estimator, Chao1, Observe, Pielow, Shannon, and Simpson), beta diversity (based on PCoA), microbial composition, and biomarker detection analyses (McMurdie and Holmes 2013). MaAsLin2 (v. 1.10.0) (https://huttenhower.sph.harvard.edu/maaslin/) was used to perform multivariate association analysis between microbial taxonomic profiles. The default settings of MaAsLin2 (v1.10.0) were utilised, with normalization set to ‘TSS’, transformation set to ‘LOG’, analysis method set to ‘LM’, and correction set to ‘BH’, except for the modification of max_significance to 0.05. The fixed_effects were specified as ‘Diagnosis’, ‘Age’, and ‘Gender’, whereas the random_effects were specified as the ID of each sample. The visualization of the top 47 associations with a q-value below 0.01 was conducted using the pheatmap package (v. 1.0.12) in R (RStudio, Boston, MA, USA), by transforming the obtained adjusted *p* values into minus log10 (*q* value), multiplying them with the sign (coefficient) to indicate the positive or negative correlations of a particular bacterium with specific conditions.

Microbial species were collected from the ProBio (http://probioticsdb.com/probiotic-strains/) and GlobalPPh (http://www.globalrph.com/bacterial-strains.htm) databases. This collection allowed the identification of 172 probiotics with beneficial effects and 88 bacterial species as pathogens (Brewer et al. 2019; Koh and Backhed 2020; Tao et al. 2017; Yu 2018). Venn diagrams were created using the ggvenn package (v. 0.1.9) (https://www.rdocumentation.org/packages/ggvenn/versions/0.1.9) in R (v. 4.3.2).

### XGB-IBD10 construction

To construct XGB-IBD10, 70% of the 9 cohorts were used, including 1,638, 220, 117, 48, 1,557, 1,207, 111, 362, and 1,537 samples from the HMP2 (included 132 participants (105 with IBD and 27 without IBD) and collected a total of 1,638 samples over approximately one year, at 24 different time points), NIHMS1510763, He et al. 2017, Liguori et al. 2016, ERP016515, SRP165757, ERP015692, SRP057027, and SRP125127 cohorts, respectively (Diederen et al. 2020; Doherty et al. 2018; Franzosa et al. 2019; He et al. 2017; Lewis et al. 2017; Liguori et al. 2016; Lloyd-Price et al. 2019; Schirmer et al. 2018; Turpin et al. 2020). Specifically, these data were used for training and validation, whereas the remaining 30% were used for testing. The abundances of microbial species with values > 99.9% and 0.1% quartiles were replaced by the 99.9% and 0.1% quartiles, respectively. Before feeding the raw features into machine learning models, it is imperative to apply the min–max method to scale and translate each feature individually, thereby guaranteeing that they fall within the range of 0~1. To address the data imbalance in the dataset, the SMOTE function of the imbalanced-learn package (v. 0.6.2) (https://imbalanced-learn.org/stable/) was used for oversampling. The top 1,000 variable species based on the microbial genera identified in HMP2, NIHMS1510763, and the other cohorts were selected using the var function in R (version 4.2.1 [RStudio]) (https://www.r-project.org/). First, we organised our dataset such that each row represented a different species and each column corresponded to different samples. Next, the var function was applied to each species (row) to calculate the variance in abundance across samples. Finally, we ranked the species in descending order, considering their variance values, and the top species were selected.

The XGB classifier library in Python (RStudio) was utilized to construct the XGBoost (v. 0.90) (https://xgboost.readthedocs.io/en/latest/python/) algorithm-based classification. The XGBoost classifier was fitted using default parameters (Chen and Guestrin 2016). SHAP (v. 0.32.1) (https://shap.readthedocs.io/en/latest/) was used to filter the top species with SHAP values, including the top species (2, 5, 10, 20, 40, 80, and 160). The SHAP values were calculated considering the training set. In cases of uneven class distribution, tenfold cross-validation was conducted to determine various performance metrics, such as accuracy, AUC, specificity, sensitivity, recall, precision, F1 score, and kappa. The model was validated using metagenomic sequencing data from 181 stool samples.

### Data standardization

To analyze the microbial sequencing data, KneadData (v. 0.10.0) software (http://huttenhower.sph.harvard.edu/kneaddata) was used to filter the human genome present in the sequencing reads and produce alignment statistics. Additionally, MetaPhlAn4 (v. 4.0.2) (https://huttenhower.sph.harvard.edu/metaphlan/) was used to generate taxonomic profiles from the shotgun metagenomes (Beghini et al. 2021). To normalize the microbial sequencing data, the coverage of the clade was standardized across all identified clades through the MetaPhlAn4’s built-in processes, enabling the determination of the relative abundance of each taxon, as previously explained (Beghini et al. 2021). The 16S data were analyzed using the quantitative insights into the microbial ecology (QIIME v1.8.0 [GitHub, San Francisco, CA, USA]) pipeline with default parameters (Caporaso et al. 2010). Operational taxonomic units (OTUs) were clustered at a 97% threshold against the Greengenes database v.13_8 (McDonald et al. 2012). OTU abundance tables were generated, representing the frequency of each OTU across samples. Only OTUs annotated to the species level (e.g., *Akkermansia muciniphila*) were retained for downstream analysis. OTUs with ambiguous labels (e.g., “uncultured bacterium”) or higher taxonomic ranks (e.g., genus Bacteroides) were excluded. The retained OTU abundance table, containing exclusively species-annotated entries, was used as the species-level profile. Samples with < 3,000 reads after quality filtering were excluded from the analysis. The OTU abundances were normalized based on known or predicted 16S copy numbers (Liguori et al. 2016; Schirmer et al. 2018). Prior to inputting the coverage of the clade from processed microbial sequencing data or OTU abundances from 16S sequencing data into machine learning models, it is essential to employ min–max normalization to ensure that each feature is individually scaled and translated, thereby ensuring that they are confined within the range of 0 to 1 (Hu et al. 2020).

### Construction of IBD models based on the metagenomic or 16S sequencing data

As mentioned above, we developed and evaluated the model using 2,337 metagenomic sequencing samples (control: 565; IBD: 1.772) obtained from HMP2, NIHMS1510763, SRP057027, and He et al. 2017 cohorts. For the 16S data, we developed and evaluated the model using 4,460 16S sequencing samples, consisting of 1,582 control samples and 2,878 samples from patients with IBD collected from SRP165757, SRP125127, ERP016515, ERP015692, and Liguori et al. 2016 cohorts. The performance of the model was validated using external data obtained from MC-IBD cohort.

### Construction of UC and CD classification models

A thorough microbial signature analysis was conducted by employing a feature selection method based on the top SHAP. HMP2 and NIHMS1510763 datasets contained UC and CD data, whereas the SRP057027 and He et al. 2017 cohorts solely contained data on CD and controls, respectively. Consequently, HMP2 and NIHMS1510763 were employed as training and testing datasets, respectively, for the classification of UC and CD. To increase the adaptability of the model to more datasets, we calculated var values based on four HMP2, NIHMS1510763, SRP057027, and He et al. 2017 metagenomic cohorts and selected the top 250 microbial species with the highest abundance variation. Subsequently, we calculated the SHAP values for the model. We used 103 samples (53 UC and 50 CD) for external validation, as 4 of the 107 IBD samples had no clear classification of UC and CD.

### Comparison of XGB-IBD10 with other machine learning and deep learning-based methods

To improve the accuracy and efficiency of the machine learning models for MC-IBD cohort, we employed various methods, including parametric techniques, such as logistic regression and Naïve Bayes, and nonparametric approaches, such as random forest, decision tree, SVM (Radial Kernel), and k-nearest neighbor. As described above, the classification models were constructed using XGBoost, random forest, k-nearest neighbors, decision tree, logistic regression, SVM, and Naïve Bayes algorithms with a 10-species signature. Each model was trained and validated considering methods used in a previous analysis.

To enhance the accuracy and efficiency of machine learning models on MC-IBD cohort, we utilized deep learning techniques, including the AE, DAE, SVAE, and CNN algorithms (Guo et al. 2020; Lee et al. 2020; Leng et al. 2022; Mohaiminul Islam et al. 2020; Seal et al. 2020). The AE, DAE, and SVAE models consisted of an input layer, two hidden layers, and a decoding layer. The input layer contained microbial taxonomic abundance data, whereas the output from the decoding layer was fed into an MLP-based model for accurate prediction and model building. Additionally, the CNN model employed Conv1D and MaxPooling1D layers to process the microbial taxonomic abundance data. The output predictions from the CNN model were passed to the MLP for model construction and testing. Each model was trained and validated using the previously described methodology (XGB-IBD10 construction part).

### Comparison of XGB-IBD10 with other species signature-based methods

We constructed machine-learning models using different species signatures to compare their accuracy and efficiencies on external validation data. The XGBoost feature selection algorithm was used to select the top 10 species with the highest SHAP values from a pool of 1,000 variable species. Additionally, we constructed classification models using other signatures, including RISK-IBD21 (consisting of 21 dominant species in CD) (Gevers et al. 2014)., UMCG IBD-IBD175 (with 175 species statistically significant in the IBD group) (Vich Vila et al. 2018), HMP2-IBD100 (comprising the top 100 differentially abundant species (smallest *P*-values) of the IBD group) (Lloyd-Price et al. 2019), and PRISM-IBD100 (containing the top 100 IBD-microbial species associations (smallest *P*-values) of the IBD group) (Franzosa et al. 2019) (Franzosa et al. 2019; Gevers et al. 2014; Lloyd-Price et al. 2019; Vich Vila et al. 2018). External signature-based methods (RISK-IBD21, UMCG IBD-175, HMP2-IBD100, PRISM-IBD100) were implemented using species lists directly sourced from their respective original studies. No additional filtering or ranking was applied to ensure fidelity to the published methodologies. Each model was trained and validated using the methods described in our previous study.

### Comparison of cohorts from different geographic origins with the XGB-IBD10 cohort

We constructed AI classification models for the Chinese (117 samples from He et al. (2017)) and cohorts from other regions (approximately 70% of the 6,680 samples were sourced outside China). The other cohort included 1,638, 220, 48, 1,557, 1,207, 111, 362, and 1,537 samples from HMP2, NIHMS1510763, Liguori et al. 2016, ERP016515, SRP165757, ERP015692, SRP057027, and SRP125127, respectively. To assess the performance of our models, we employed tenfold cross-validation and evaluated metrics such as accuracy, AUC, specificity, sensitivity, recall, precision, F1 score, and kappa. Furthermore, we tested XGB-IBD10 using external validation data from 181 collected samples.

## Supplementary Information

Supplementary Material 1. Fig. S1 The definition of Accuracy, Specific, Recall, Precision, F1, and Kappa. Fig. S2 Analysis of feature importance based on SHAP values. Fig. S3 Distribution of Clinic data. Table S1 The SHAP value of each specie in the top 1000 variable species-based IBD classification model. Table S2 The SHAP value of each specie in 10-species signature-based IBD classification model. Table S3 The SHAP value of each specie in the top 250 variable species-based UC/CD classification model. Table S4 The SHAP value of each specie in 5-species signature-based UC/CD classification model. Table S5 The clinical data of 107 IBD patients. Table S6 The SHAP value (above 0) of each feature in metagenomic and clinic data-based classification model.

## Data Availability

The metagenomic sequence data are deposited in the Genome Sequence Archive (GSA) under bioProject PRJCA017134 with the accession number of HRA004640 (https://ngdc.cncb.ac.cn/gsa-human/browse/HRA004640). The metagenomic sequence data is presently accessible to all individuals with the authorization of the Data Access Committee (DAC), and we promptly provided access to the data without DAC authorization prior to publishing the article online. All other data generated or analyzed during the course of this study are fully documented and available in the published article and its supplementary information files.

## Abbreviations

AUC: Area under the curve
CD: Crohn’s disease
UC: Ulcerative colitis
IBD: Inflammatory bowel disease
HMP2: The Integrative Human Microbiome Project
ROC: Receiver operating characteristic
SHAP: SHapley Additive exPlanations
XGBoost: XGBoost Extreme Gradient Boosting
XGB-IBD10: The 10-species signature-based XGBoost classification model
UMCG: The University Medical Center of Groningen IBD
PRISM: The Prospective Registry in IBD Study at MGH (the Massachusetts General Hospital)
