# Supplementary material for: Identification of a 10-species microbial signature of inflammatory bowel disease by machine learning and external validation
Source: Cell Regen. 2025 Jul 14;14:32. doi: 10.1186/s13619-025-00246-w (PMC12259524; doi:10.1186/s13619-025-00246-w)
Supplement: Supplementary file 1 — Supplementary Material 1. Fig. S1 The definition of Accuracy, Specific, Recall, Precision, F1, and Kappa. Fig. S2 Analysis of feature importance based on SHAP values. Fig. S3 Distribution of Clinic data. Table S1 The SHAP value of each specie in the top 1000 variable species-based IBD classification model. Table S2 The SHAP value of each specie in 10-species signature-based IBD classification model. Table S3 The SHAP value of each specie in the top 250 variable species-based UC/CD classification model. Table S4 The SHAP value of each specie in 5-species signature-based UC/CD classification model. Table S5 The clinical data of 107 IBD patients. Table S6 The SHAP value (above 0) of each feature in metagenomic and clinic data-based classification model. [file 13619_2025_246_MOESM1_ESM.doc]

Supplementary information

**Identification of a 10-species microbial signature of inflammatory bowel disease by machine learning and external validation**

Shicheng Yu^1, #^, Jun Li^2, #^, Zhaofeng Ye^3^, Mengxian Zhang^4^, Xiaohua Guo^2^, Xu Wang^1^, Liansheng Liu^1^, Yalong Wang^1^, Xin Zhou^2^, Wei Fu^2^, Michael Q Zhang^3^ and Ye-Guang Chen^1,4,5, *^

**Supplementary Figure 1 to 3**

**Supplementary Table 1 to 6**


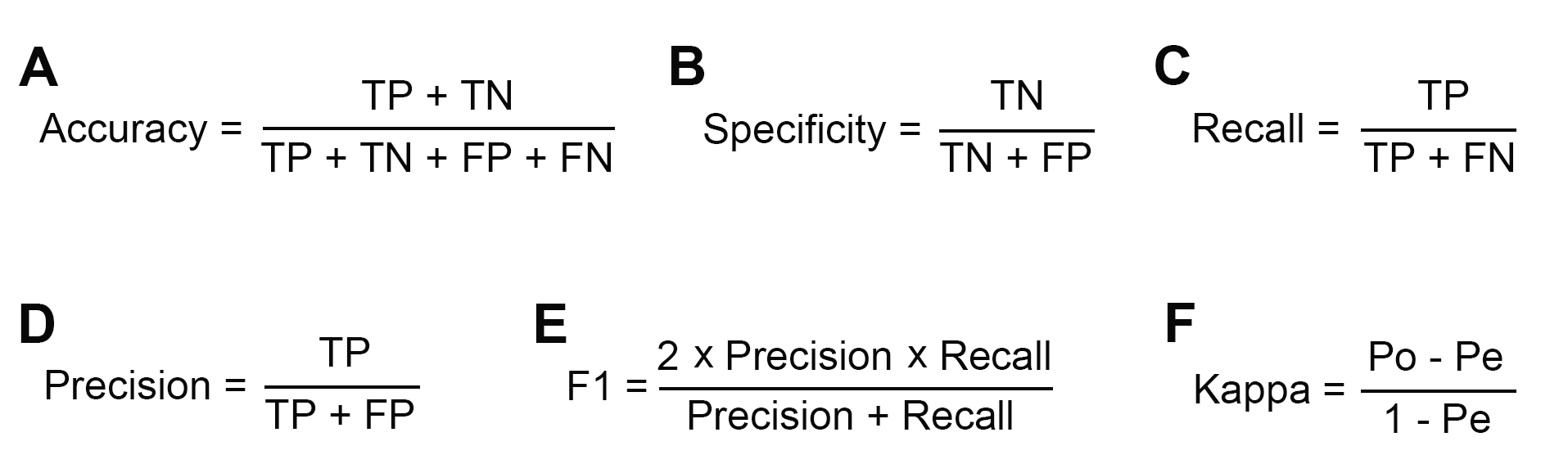


**Supplementary Figure 1** The definition of Accuracy (**A**), Specific (**B**), Recall (**C**), Precision (**D**), F1 (**E**), and Kappa (**F**) is provided by Equation. TP: True Positive; TN: True Negative; FP: False Positive; FN: False Negative. Po refers to the empirical probability of agreement on the label assigned to any sample (also known as the observed agreement ratio), and Pe represents the expected agreement when both annotators assign labels randomly. To estimate Pe, a per-annotator empirical prior over the class labels is used.

**
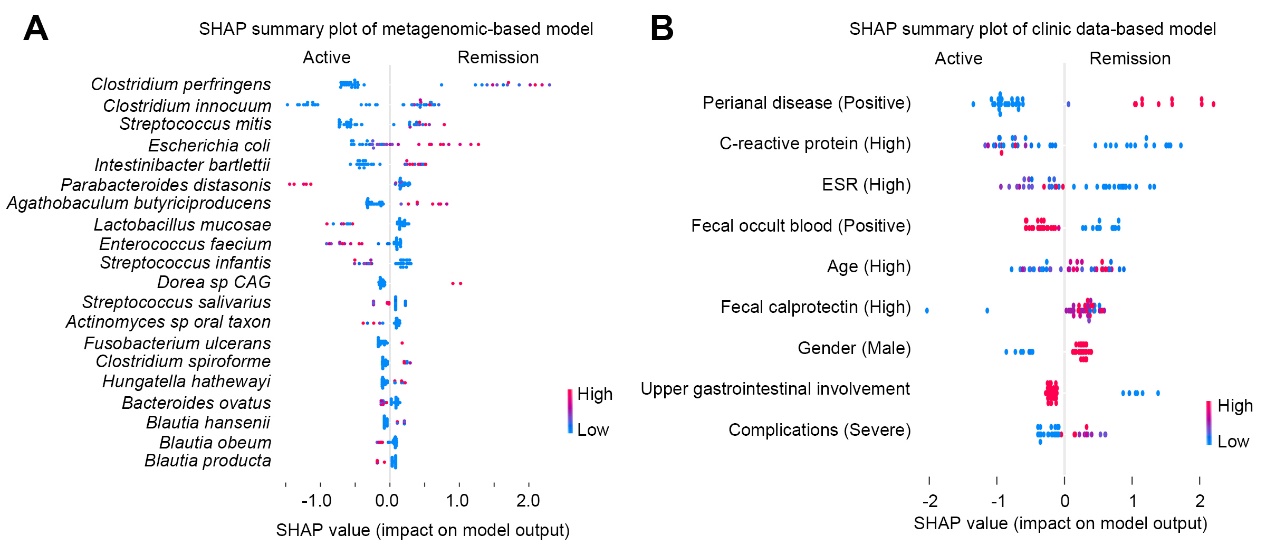
**

**Supplementary Figure 2 Analysis of feature importance based on SHAP values**. (**A**) SHAP value plot of top 20 important species of metagenomic data-based model. (**B**) SHAP value plot of top 9 important species of clinical data-based model.

**
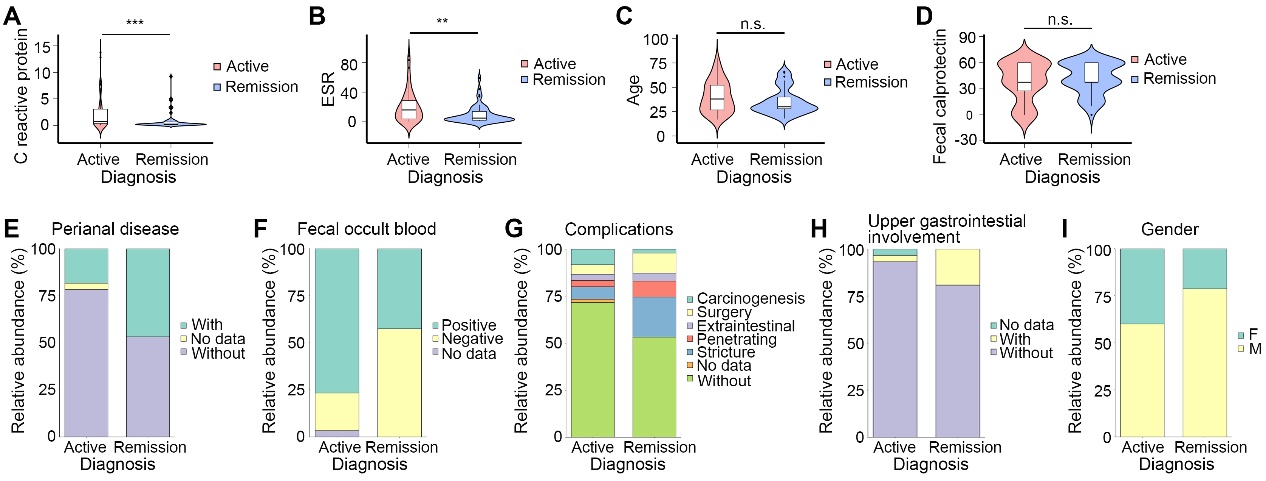
**

**Supplementary Figure 3 Distribution of Clinic data. (A-D)** The violin plots show the ratio of C reactive protein (**A**), ESR (erythrocyte sedimentation rate) (**B**), Age (**C**), Fecal calprotectin (**D**) in active and remission samples. **(E-I)** Bar plots show the ratio of perianal disease (**E**), fecal occult blood (**F**), complications (**G**), upper gastrointestinal involvement (**H**), and gender (**I**) in active and remission samples. Significance for (**A**-**D**) is calculated by Wilcoxon rank-sum test. n.s., not significant, **P* ≤ 0.05, ***P* < 0.01, ****P* < 0.001.

**Supplementary Table 1** The SHAP value of each specie in the top 1000 variable species-based IBD classification model.

| Number | The microbial species | SHAP values | Number | The microbial species | SHAP values |
| --- | --- | --- | --- | --- | --- |
| 1 | *Dorea formicigenerans* | 0.7115438 | 2 | *Fusicatenibacter saccharivorans* | 0.4213854 |
| 3 | *Anaerotruncus rubiinfantis* | 0.4151791 | 4 | *Oscilibacter massiliensis* | 0.4151237 |
| 5 | *Akkermansia muciniphila* | 0.2200305 | 6 | *Alistipes finegoldii* | 0.2123504 |
| 7 | *Ruminococcus bromii* | 0.1904767 | 8 | *Collinsella aerofaciens* | 0.1695364 |
| 9 | *Clostridioides difficile* | 0.1234047 | 10 | *Eubacterium oxidoreducens* | 0.1100903 |
| 11 | *Ruminococcus torques* | 0.1041221 | 12 | *Dialister invisus* | 0.0811312 |
| 13 | *Escherichia coli* | 0.0808322 | 14 | *Proteobacteria bacterium CAG 139* | 0.073073 |
| 15 | *Alistipes sp. AL-1* | 0.072158 | 16 | *Roseburia inulinivorans* | 0.064511 |
| 17 | *Clostridium sp.* | 0.0643371 | 18 | *Flavonifractor plautii* | 0.0593115 |
| 19 | *Bacteroides ovatus* | 0.0551763 | 20 | *Bisgaard Taxon 7* | 0.0551086 |
| 21 | *Erysipelatoclostridium sp. SNUG30386* | 0.0475953 | 22 | *Bacteroides fragilis* | 0.0449496 |
| 23 | *Dialister pneumosintes* | 0.0448934 | 24 | *Bifidobacterium animalis* | 0.0448585 |
| 25 | *Bacteroides helcogenes* | 0.0432128 | 26 | *Neglecta timonensis* | 0.0403293 |
| 27 | *Roseburia faecis* | 0.0379385 | 28 | *Blautia hydrogenotrophica* | 0.0370757 |
| 29 | *Bacteroides uniformis* | 0.0363021 | 30 | *Dialister propionicifaciens* | 0.0348347 |
| 31 | *Butyricimonas synergistica* | 0.0342247 | 32 | *Ruminococcus lactaris* | 0.0314663 |
| 33 | *Blautia obeum* | 0.0303158 | 34 | *Clostridium leptum* | 0.0296088 |
| 35 | *Blautia massiliensis* | 0.0294258 | 36 | *Dialister sp. oral clone MCE7 134* | 0.0260302 |
| 37 | *Collinsella ihuae* | 0.0255783 | 38 | *Bacteroides eggerthii* | 0.0232271 |
| 39 | *Butyricicoccus faecihominis* | 0.0228036 | 40 | *Coprococcus comes* | 0.02252 |
| 41 | *Anaerotruncus colihominis* | 0.0203246 | 42 | *Alistipes shahii* | 0.0199444 |
| 43 | *Odoribacter splanchnicus* | 0.0198203 | 44 | *Bifidobacterium adolescentis* | 0.0194402 |
| 45 | *Roseburia hominis* | 0.0188379 | 46 | *Alistipes putredinis* | 0.0184875 |
| 47 | *Dysgonomonas sp 37 18* | 0.0176739 | 48 | *Coprococcus eutactus* | 0.0172649 |
| 49 | *Bifidobacterium saguini* | 0.0160687 | 50 | *Veillonella atypica* | 0.0160037 |
| 51 | *Firmicutes bacterium CAG 83* | 0.0158882 | 52 | *Dorea longicatena* | 0.0154008 |
| 53 | *Marvinbryantia formatexigens* | 0.0147733 | 54 | *Prevotella sp 885* | 0.0145394 |
| 55 | *Parabacteroides goldsteinii* | 0.0132457 | 56 | *Paraprevotella clara* | 0.0130436 |
| 57 | *Lachnospiraceae bacterium TF01-11* | 0.0129409 | 58 | *Bifidobacterium longum* | 0.0116856 |
| 59 | *Ruminococcus sp. Marseille-P328* | 0.0115008 | 60 | *Bacteroides sp CAG 144* | 0.0109155 |
| 61 | *Clostridium symbiosum* | 0.0096638 | 62 | *Bacteroides galacturonicus* | 0.0092383 |
| 63 | *Clostridium sp. Marseille-P2415* | 0.0085751 | 64 | *Eubacterium eligens* | 0.0085277 |
| 65 | *Parasutterella excrementihominis* | 0.0072677 | 66 | *Lactonifactor sp. Marseille-P3743* | 0.0071179 |
| 67 | *Parabacteroides distasonis* | 0.0068907 | 68 | *Gemmiger formicilis* | 0.0065016 |
| 69 | *Roseburia sp CAG 182* | 0.0064881 | 70 | *Clostridium sp CAG 58* | 0.006474 |
| 71 | *Veillonella magna* | 0.006176 | 72 | *Clostridium sp CAG 242* | 0.0058 |
| 73 | *Bacteroides finegoldii* | 0.0054382 | 74 | *Negativibacillus massiliensis* | 0.0050575 |
| 75 | *Bacteroides caccae* | 0.0048499 | 76 | *Eubacterium rectale* | 0.0047814 |
| 77 | *Streptococcus parasanguinis* | 0.0043853 | 78 | *Lactobacillus rogosae* | 0.0039863 |
| 79 | *Eubacterium sp CAG 251* | 0.0039372 | 80 | *Bacteroides nordii* | 0.0035879 |
| 81 | *Streptococcus salivarius* | 0.0034596 | 82 | *Bacteroides stercoris* | 0.0034405 |
| 83 | *Ruminococcus sp CAG 330* | 0.0032844 | 84 | *Ruthenibacterium lactatiformans* | 0.0032351 |
| 85 | *Ruminococcus sp.* | 0.0032045 | 86 | *Clostridium sp CAG 299* | 0.0032037 |
| 87 | *Megasphaera sp DISK 18* | 0.0025826 | 88 | *Clostridium sp CAG 167* | 0.0023879 |
| 89 | *Clostridia bacterium UC5.1-2D9* | 0.0021923 | 90 | *Faecalibacterium prausnitzii* | 0.0021439 |
| 91 | *Bacteroides cellulosilyticus* | 0.0021341 | 92 | *Fusobacterium varium* | 0.0016987 |
| 93 | *Coprobacter sp* | 0.0013308 | 94 | *Eubacterium sp CAG 38* | 0.0012751 |
| 95 | *Streptococcus mutans* | 0.0010327 | 96 | *Dialister sp. S7D* | 0.0009684 |
| 97 | *Anaerococcus sp. S138* | 0.0009528 | 98 | *Anaerostipes hadrus* | 0.0007852 |
| 99 | *Colidextribacter massiliensis* | 0.0007433 | 100 | *Ruminococcus gnavus* | 0.0006656 |
| 101 | *Parabacteroides merdae* | 0.0005885 | 102 | *Eubacterium siraeum* | 0.0005855 |
| 103 | *Prevotella buccalis* | 0.0005551 | 104 | *Firmicutes bacterium CAG 145* | 0.0005307 |
| 105 | *Clostridiales bacterium 42 27* | 0.0005251 | 106 | *Agathobaculum butyriciproducens* | 0.0004867 |
| 107 | *Clostridium sp. AT4* | 0.0003554 | 108 | *Raoultibacter massiliensis* | 0.0003395 |
| 109 | *Bifidobacterium bifidum* | 0.0002786 | 110 | *Acetitomaculum ruminis* | 0 |
| 111 | *Acetivibrio ethanolgignens* | 0 | 112 | *Acetobacteraceae bacterium* | 0 |
| 113 | *Acetobacteraceae bacterium MP03* | 0 | 114 | *Acholeplasma pleciae* | 0 |
| 115 | *Acholeplasmatales bacterium canine oral taxon 316* | 0 | 116 | *Acholeplasmatales bacterium canine oral taxon 375* | 0 |
| 117 | *Acholeplasmatales bacterium feline oral taxon 303* | 0 | 118 | *Acidaminococcus fermentans* | 0 |
| 119 | *Acidaminococcus intestini* | 0 | 120 | *Acidaminococcus sp. DJF RP55* | 0 |
| 121 | *Acidobacteria bacterium CB 286367* | 0 | 122 | *Acidobacteria bacterium LX3* | 0 |
| 123 | *Acinetobacter baumannii* | 0 | 124 | *Acinetobacter calcoaceticus* | 0 |
| 125 | *Acinetobacter junii* | 0 | 126 | *Acinetobacter sp. IrC1* | 0 |
| 127 | *Acinetobacter ursingii* | 0 | 128 | *Actinobaculum massiliense* | 0 |
| 129 | *Actinomyces cardiffensis* | 0 | 130 | *Actinomyces coleocanis* | 0 |
| 131 | *Actinomyces graevenitzii* | 0 | 132 | *Actinomyces odontolyticus* | 0 |
| 133 | *Actinomyces turicensis* | 0 | 134 | *Actinoplanes couchii* | 0 |
| 135 | *Actinoplanes sp. 80820* | 0 | 136 | *Adhaeribacter aquaticus* | 0 |
| 137 | *Aegilops tauschii* | 0 | 138 | *Aequorivita capsosiphonis* | 0 |
| 139 | *Aeromonas hydrophila* | 0 | 140 | *Agaricicola taiwanensis* | 0 |
| 141 | *Agathobacter ruminis* | 0 | 142 | *Aggregatibacter actinomycetemcomitans* | 0 |
| 143 | *Aggregatibacter aphrophilus* | 0 | 144 | *Aggregatibacter segnis* | 0 |
| 145 | *Alcaligenes sp. I 0.0* | 0 | 146 | *Alistipes ihumii* | 0 |
| 147 | *Alistipes indistinctus* | 0 | 148 | *Alistipes inops* | 0 |
| 149 | *Alistipes onderdonkii* | 0 | 150 | *Alistipes sp. RMA 9912* | 0 |
| 151 | *Alkaliphilus sp. LacT* | 0 | 152 | *Allisonella histaminiformans* | 0 |
| 153 | *Allobaculum stercoricanis* | 0 | 154 | *Allohahella marinimesophila* | 0 |
| 155 | *Alloprevotella tannerae* | 0 | 156 | *Alphaproteobacteria bacterium canine oral taxon 081* | 0 |
| 157 | *Amborella trichopoda* | 0 | 158 | *Anaerobium acetethylicum* | 0 |
| 159 | *Anaerococcus mediterraneensis* | 0 | 160 | *Anaerococcus octavius* | 0 |
| 161 | *Anaerococcus prevotii* | 0 | 162 | *Anaerococcus sp. Marseille-P2143 0.794000000000096* | 0 |
| 163 | *Anaerofilum sp. Marseille-P3374* | 0 | 164 | *Anaeromassilibacillus senegalensis* | 0 |
| 165 | *Anaeromassilibacillus sp. Marseille-P3371* | 0 | 166 | *Anaeromassilibacillus sp An250* | 0 |
| 167 | *Anaerorhabdus furcosa* | 0 | 168 | *Anaerosporobacter mobilis* | 0 |
| 169 | *Anaerostipes butyraticus* | 0 | 170 | *Anaerostipes caccae* | 0 |
| 171 | *Anaerostipes rhamnosivorans* | 0 | 172 | *Anaerostipes sp. IE4* | 0 |
| 173 | *Anaerotignum lactatifermentans* | 0 | 174 | *Anaerotruncus sp CAG 528* | 0 |
| 175 | *Angelakisella massiliensis* | 0 | 176 | *Aquaspirillum sp. feline oral taxon 082* | 0 |
| 177 | *Aquihabitans daechungensis* | 0 | 178 | *Aquimonas sp. enrichment culture clone 03SUJ1* | 0 |
| 179 | *Arabidopsis thaliana (thale cress)* | 0 | 180 | *Arcobacter butzleri* | 0 |
| 181 | *Armatimonadetes bacterium 55-13* | 0 | 182 | *Asticcacaulis solisilvae* | 0 |
| 183 | *Atopostipes sp. 17.2 AW* | 0 | 184 | *Auxenochlorella protothecoides* | 0 |
| 185 | *Azotobacter chroococcum* | 0 | 186 | *Bacillus cereus* | 0 |
| 187 | *Bacteriovorax sp. EPA* | 0 | 188 | *Bacteroidaceae bacterium DJF B220* | 0 |
| 189 | *Bacteroidales bacterium 36-12* | 0 | 190 | *Bacteroidales bacterium 55 9* | 0 |
| 191 | *Bacteroidales bacterium H4* | 0 | 192 | *Bacteroides acidifaciens* | 0 |
| 193 | *Bacteroides caecigallinarum* | 0 | 194 | *Bacteroides clarus* | 0 |
| 195 | *Bacteroides coprocola* | 0 | 196 | *Bacteroides coprophilus* | 0 |
| 197 | *Bacteroides dorei* | 0 | 198 | *Bacteroides faecis* | 0 |
| 199 | *Bacteroides faecis CAG 32* | 0 | 200 | *Bacteroides fluxus* | 0 |
| 201 | *Bacteroides gallinaceum* | 0 | 202 | *Bacteroides gallinarum* | 0 |
| 203 | *Bacteroides ihuae* | 0 | 204 | *Bacteroides intestinalis* | 0 |
| 205 | *Bacteroides massiliensis* | 0 | 206 | *Bacteroides oleiciplenus* | 0 |
| 207 | *Bacteroides paurosaccharolyticus* | 0 | 208 | *Bacteroides pectinophilus* | 0 |
| 209 | *Bacteroides plebeius* | 0 | 210 | *Bacteroides pyogenes* | 0 |
| 211 | *Bacteroides rodentium* | 0 | 212 | *Bacteroides salyersiae* | 0 |
| 213 | *Bacteroides sartorii* | 0 | 214 | *Bacteroides sp.* | 0 |
| 215 | *Bacteroides sp. AN 5745* | 0 | 216 | *Bacteroides sp. DSM 12148* | 0 |
| 217 | *Bacteroides sp. Marseille-P3108* | 0 | 218 | *Bacteroides sp. Marseille-P3208T* | 0 |
| 219 | *Bacteroides sp. PFB2-14* | 0 | 220 | *Bacteroides sp. PH5-1* | 0 |
| 221 | *Bacteroides sp. PH5-19* | 0 | 222 | *Bacteroides sp. R6* | 0 |
| 223 | *Bacteroides sp. S427* | 0 | 224 | *Bacteroides sp. WA1* | 0 |
| 225 | *Bacteroides sp. feline oral taxon 308* | 0 | 226 | *Bacteroides sp 43 108* | 0 |
| 227 | *Bacteroides sp CAG 633* | 0 | 228 | *Bacteroides thetaiotaomicron* | 0 |
| 229 | *Bacteroides vulgatus* | 0 | 230 | *Bacteroides xylanisolvens* | 0 |
| 231 | *Bacteroidetes bacterium CHC2* | 0 | 232 | *Bacteroidia bacterium canine oral taxon 387* | 0 |
| 233 | *Bacteroidia bacterium feline oral taxon 115* | 0 | 234 | *Bacteroidia bacterium feline oral taxon 312* | 0 |
| 235 | *Bariatricus massiliensis* | 0 | 236 | *Barnesiella intestinihominis* | 0 |
| 237 | *Bdellovibrio bacteriovorus* | 0 | 238 | *Bdellovibrio sp. SKB1291214* | 0 |
| 239 | *Bdellovibrionales bacterium GWC1 52 8* | 0 | 240 | *Bdellovibrionales bacterium RIFCSPHIGHO2 01 FULL 40 29* | 0 |
| 241 | *Beduini massiliensis* | 0 | 242 | *Beduinibacterium massiliense* | 0 |
| 243 | *Bergeyella zoohelcum* | 0 | 244 | *Bernardetia litoralis* | 0 |
| 245 | *Bibersteinia trehalosi* | 0 | 246 | *Bifidobacteriaceae genomosp. C1* | 0 |
| 247 | *Bifidobacterium aerophilum* | 0 | 248 | *Bifidobacterium angulatum* | 0 |
| 249 | *Bifidobacterium avesanii* | 0 | 250 | *Bifidobacterium breve* | 0 |
| 251 | *Bifidobacterium catenulatum* | 0 | 252 | *Bifidobacterium choerinum* | 0 |
| 253 | *Bifidobacterium commune* | 0 | 254 | *Bifidobacterium dentium* | 0 |
| 255 | *Bifidobacterium pseudocatenulatum* | 0 | 256 | *Bifidobacterium pseudolongum* | 0 |
| 257 | *Bifidobacterium reuteri* | 0 | 258 | *Bifidobacterium sp. 113* | 0 |
| 259 | *Bifidobacterium sp. MC 10* | 0 | 260 | *Bifidobacterium sp. MRM 8.19* | 0 |
| 261 | *Bifidobacterium sp. MRM 9.03* | 0 | 262 | *Bifidobacterium tsurumiense* | 0 |
| 263 | *Bifissio spartinae* | 0 | 264 | *Bilophila sp. S375* | 0 |
| 265 | *Bilophila wadsworthia* | 0 | 266 | *Bisgaard Taxon 10* | 0 |
| 267 | *Blautia caecimuris* | 0 | 268 | *Blautia hansenii* | 0 |
| 269 | *Blautia producta* | 0 | 270 | *Blautia sp. Marseille-P3201T* | 0 |
| 271 | *Blautia sp. Marseille-P3387* | 0 | 272 | *Blautia sp. N6H1-15* | 0 |
| 273 | *Blautia sp. NOVO1-10* | 0 | 274 | *Blautia sp. YL58* | 0 |
| 275 | *Blautia sp. canine oral taxon 143* | 0 | 276 | *Blautia sp CAG 257* | 0 |
| 277 | *Blautia stercoris* | 0 | 278 | *Blautia wexlerae* | 0 |
| 279 | *Brenneria alni* | 0 | 280 | *Brevundimonas sp. ALBL 122* | 0 |
| 281 | *Buchnera aphidicola* | 0 | 282 | *Bulleidia extructa* | 0 |
| 283 | *Burkholderia sp. K4410.MGS-135* | 0 | 284 | *Burkholderiales bacterium X4* | 0 |
| 285 | *Butyricicoccus pullicaecorum* | 0 | 286 | *Butyricimonas virosa* | 0 |
| 287 | *Butyrivibrio crossotus* | 0 | 288 | *Butyrivibrio sp CAG 318* | 0 |
| 289 | *Campylobacter concisus* | 0 | 290 | *Campylobacter hominis* | 0 |
| 291 | *Campylobacter rectus* | 0 | 292 | *Campylobacter ureolyticus* | 0 |
| 293 | *Candidatus Hepatoplasma crinochetorum* | 0 | 294 | *Candidatus Lumbricincola sp. Lr-C2* | 0 |
| 295 | *Candidatus Melainabacteria bacterium MEL.A1* | 0 | 296 | *Candidatus Metachlamydia lacustris* | 0 |
| 297 | *Candidatus Methanomassiliicoccus intestinalis* | 0 | 298 | *Candidatus Portiera aleyrodidarum* | 0 |
| 299 | *Candidatus Schneideria nysicola* | 0 | 300 | *Candidatus Soleaferrea massiliensis* | 0 |
| 301 | *Candidatus Stoquefichus sp KLE1796* | 0 | 302 | *Candidatus Sulcia muelleri* | 0 |
| 303 | *Candidatus Tremblaya phenacola* | 0 | 304 | *Candidatus Tremblaya princeps* | 0 |
| 305 | *Cannabis sativa (hemp)* | 0 | 306 | *Capnocytophaga canimorsus* | 0 |
| 307 | *Capnocytophaga sp. KC07084* | 0 | 308 | *Capnocytophaga sp. canine oral taxon 329* | 0 |
| 309 | *Caproiciproducens galactitolivorans 0.0* | 0 | 310 | *Capsicum annuum* | 0 |
| 311 | *Capsicum annuum var. glabriusculum* | 0 | 312 | *Catabacter hongkongensis* | 0 |
| 313 | *Catenibacterium mitsuokai* | 0 | 314 | *Catonella morbi* | 0 |
| 315 | *Catonella sp. canine oral taxon 025* | 0 | 316 | *Catonella sp. oral clone AH153 0.0* | 0 |
| 317 | *Caulobacter sp. P-7Y* | 0 | 318 | *Cellulosilyticum lentocellum* | 0 |
| 319 | *Chishuiella changwenlii* | 0 | 320 | *Chlorosarcina brevispinosa* | 0 |
| 321 | *Christensenella massiliensis* | 0 | 322 | *Christensenella timonensis* | 0 |
| 323 | *Citrobacter amalonaticus* | 0 | 324 | *Citrobacter freundii* | 0 |
| 325 | *Citrobacter sp.* | 0 | 326 | *Citrobacter sp. GIST-MRh35* | 0 |
| 327 | *Citrobacter werkmanii* | 0 | 328 | *Citrobacter youngae* | 0 |
| 329 | *Cloacibacillus evryensis* | 0 | 330 | *Cloacibacterium sp. canine oral taxon 320* | 0 |
| 331 | *Clostridia bacterium UC5.1-2E3* | 0 | 332 | *Clostridia bacterium enrichment culture clone WSC-26* | 0 |
| 333 | *Clostridiaceae bacterium DJF VR07* | 0 | 334 | *Clostridiaceae bacterium DJF VR09* | 0 |
| 335 | *Clostridiaceae bacterium DJF VR76* | 0 | 336 | *Clostridiaceae bacterium NML 060002* | 0 |
| 337 | *Clostridiaceae bacterium SH032* | 0 | 338 | *Clostridiales bacterium 21-4c* | 0 |
| 339 | *Clostridiales bacterium 30-4c* | 0 | 340 | *Clostridiales bacterium 40-4c* | 0 |
| 341 | *Clostridiales bacterium 41-2a* | 0 | 342 | *Clostridiales bacterium CHKCI006* | 0 |
| 343 | *Clostridiales bacterium CIEAF 020* | 0 | 344 | *Clostridiales bacterium DJF B152* | 0 |
| 345 | *Clostridiales bacterium Firm 08* | 0 | 346 | *Clostridiales bacterium KA00134* | 0 |
| 347 | *Clostridiales bacterium Marseille-P2846* | 0 | 348 | *Clostridiales bacterium NK3B98* | 0 |
| 349 | *Clostridiales bacterium canine oral taxon 027* | 0 | 350 | *Clostridiales bacterium canine oral taxon 028* | 0 |
| 351 | *Clostridiales bacterium canine oral taxon 100* | 0 | 352 | *Clostridiales bacterium canine oral taxon 141* | 0 |
| 353 | *Clostridiales bacterium canine oral taxon 216* | 0 | 354 | *Clostridiales bacterium canine oral taxon 217* | 0 |
| 355 | *Clostridiales bacterium canine oral taxon 261* | 0 | 356 | *Clostridiales bacterium canine oral taxon 386* | 0 |
| 357 | *Clostridiales bacterium enrichment culture clone LDC-10 0.0* | 0 | 358 | *Clostridiales bacterium oral clone MCE3 9* | 0 |
| 359 | *Clostridiales bacterium oral taxon F32* | 0 | 360 | *Clostridium aldenense* | 0 |
| 361 | *Clostridium bolteae* | 0 | 362 | *Clostridium bolteae CAG 59* | 0 |
| 363 | *Clostridium bornimense* | 0 | 364 | *Clostridium butyricum* | 0 |
| 365 | *Clostridium cellulovorans* | 0 | 366 | *Clostridium chauvoei* | 0 |
| 367 | *Clostridium citroniae* | 0 | 368 | *Clostridium clostridioforme* | 0 |
| 369 | *Clostridium colicanis* | 0 | 370 | *Clostridium cylindrosporum* | 0 |
| 371 | *Clostridium disporicum* | 0 | 372 | *Clostridium innocuum* | 0 |
| 373 | *Clostridium lavalense* | 0 | 374 | *Clostridium neonatale* | 0 |
| 375 | *Clostridium paraputrificum* | 0 | 376 | *Clostridium saccharolyticum* | 0 |
| 377 | *Clostridium septicum* | 0 | 378 | *Clostridium sp. BPY5* | 0 |
| 379 | *Clostridium sp. CE6* | 0 | 380 | *Clostridium sp. Culture Jar-19* | 0 |
| 381 | *Clostridium sp. FRC Cl1 0.0* | 0 | 382 | *Clostridium sp. MT10-315-CC-82* | 0 |
| 383 | *Clostridium sp. Marseille-P299* | 0 | 384 | *Clostridium sp. Marseille-P3244* | 0 |
| 385 | *Clostridium sp. ND2* | 0 | 386 | *Clostridium sp. T2* | 0 |
| 387 | *Clostridium sp. TO-931* | 0 | 388 | *Clostridium sp. cTPY-17* | 0 |
| 389 | *Clostridium sp. cf3-PUG* | 0 | 390 | *Clostridium sp. mt5* | 0 |
| 391 | *Clostridium sp CAG 411* | 0 | 392 | *Clostridium sp CAG 590* | 0 |
| 393 | *Clostridium sp CAG 678* | 0 | 394 | *Clostridium spiroforme* | 0 |
| 395 | *Collinsella bouchesdurhonensis* | 0 | 396 | *Collinsella intestinalis* | 0 |
| 397 | *Collinsella phocaeensis* | 0 | 398 | *Collinsella sp. MS5* | 0 |
| 399 | *Collinsella sp. Marseille-P3740* | 0 | 400 | *Collinsella stercoris* | 0 |
| 401 | *Collinsella tanakaei* | 0 | 402 | *Comamonas aquatica* | 0 |
| 403 | *Comamonas sp. AM1* | 0 | 404 | *Conchiformibius steedae* | 0 |
| 405 | *Conexibacter sp.* | 0 | 406 | *Coprobacillus cateniformis* | 0 |
| 407 | *Coprobacter fastidiosus* | 0 | 408 | *Coprobacter secundus* | 0 |
| 409 | *Coprococcus catus* | 0 | 410 | *Coriobacteriaceae bacterium 68-1-3* | 0 |
| 411 | *Coriobacteriaceae bacterium CHKCI002* | 0 | 412 | *Corynebacterium accolens* | 0 |
| 413 | *Corynebacterium camporealensis* | 0 | 414 | *Corynebacterium coyleae* | 0 |
| 415 | *Corynebacterium durum* | 0 | 416 | *Corynebacterium fournierii* | 0 |
| 417 | *Corynebacterium genitalium* | 0 | 418 | *Corynebacterium glaucum* | 0 |
| 419 | *Corynebacterium mucifaciens* | 0 | 420 | *Corynebacterium pilbarense* | 0 |
| 421 | *Corynebacterium pseudogenitalium* | 0 | 422 | *Corynebacterium sp.* | 0 |
| 423 | *Corynebacterium sp. 110393* | 0 | 424 | *Corynebacterium sp. 1RN-3G* | 0 |
| 425 | *Corynebacterium sp. 2002-79006* | 0 | 426 | *Corynebacterium sp. C20* | 0 |
| 427 | *Corynebacterium sp. HMSC070H05* | 0 | 428 | *Corynebacterium sp. HMSC08F01* | 0 |
| 429 | *Corynebacterium sp. HMSC11D10* | 0 | 430 | *Corynebacterium sp. MC3* | 0 |
| 431 | *Corynebacterium sp. M 72* | 0 | 432 | *Corynebacterium sp. NG11* | 0 |
| 433 | *Corynebacterium sp. canine oral taxon 422* | 0 | 434 | *Corynebacterium tuscaniense* | 0 |
| 435 | *Corynebacterium ureicelerivorans* | 0 | 436 | *Corynebacterium vitaeruminis* | 0 |
| 437 | *Corynebacterium xerosis* | 0 | 438 | *Craurococcus roseus* | 0 |
| 439 | *Criibacterium bergeronii* | 0 | 440 | *Cronobacter dublinensis* | 0 |
| 441 | *Cronobacter turicensis* | 0 | 442 | *Cryptobacterium curtum* | 0 |
| 443 | *Culturomica massiliensis* | 0 | 444 | *Cuneatibacter caecimuris* | 0 |
| 445 | *Cutibacterium acnes* | 0 | 446 | *Cytophaga hutchinsonii* | 0 |
| 447 | *D168* | 0 | 448 | *Dakarella massiliensis* | 0 |
| 449 | *Deinococcus piscis* | 0 | 450 | *Deinococcus sp. A23.3* | 0 |
| 451 | *Deltaproteobacteria bacterium canine oral taxon 266* | 0 | 452 | *Dendrosporobacter quercicolus 1.0* | 0 |
| 453 | *Dermatophilus crocodyli* | 0 | 454 | *Desmochloris halophila* | 0 |
| 455 | *Desulfomicrobium orale* | 0 | 456 | *Desulfonispora thiosulfatigenes 0.0* | 0 |
| 457 | *Desulfonosporus sp. AAN04* | 0 | 458 | *Desulfotomaculum intricatum* | 0 |
| 459 | *Desulfotomaculum peckii* | 0 | 460 | *Desulfotomaculum sp. cs1-2* | 0 |
| 461 | *Desulfovibrio piger* | 0 | 462 | *Desulfovibrio sp. enrichment culture clone Jdgsrb011* | 0 |
| 463 | *Desulfovibrionaceae bacterium* | 0 | 464 | *Desulfovibrionaceae bacterium Marseille-P3669* | 0 |
| 465 | *Desulfovibrionales bacterium canine oral taxon 009* | 0 | 466 | *Devosia neptuniae* | 0 |
| 467 | *Devosia sp. Bzb6* | 0 | 468 | *Dialister sp. oral clone BS016* | 0 |
| 469 | *Dialister sp. oral clone FY011* | 0 | 470 | *Dialister sp CAG 357* | 0 |
| 471 | *Dialister succinatiphilus* | 0 | 472 | *Dickeya chrysanthemi* | 0 |
| 473 | *Dielma fastidiosa* | 0 | 474 | *Dientamoeba fragilis* | 0 |
| 475 | *Dioscorea nipponica* | 0 | 476 | *Dorea sp. D27* | 0 |
| 477 | *Dorea sp CAG 317* | 0 | 478 | *Drancourtella massiliensis* | 0 |
| 479 | *Dubosiella newyorkensis* | 0 | 480 | *Duodenibacillus massiliensis* | 0 |
| 481 | *Dysgonomonas gadei* | 0 | 482 | *Dysgonomonas hofstadii* | 0 |
| 483 | *Dysgonomonas sp. PH5-20* | 0 | 484 | *Dysgonomonas sp. PH5-45* | 0 |
| 485 | *Eggerthella lenta* | 0 | 486 | *Eggerthellaceae bacterium* | 0 |
| 487 | *Eikenella corrodens* | 0 | 488 | *Eikenella sp. NML02-A-017 1.46446022557265* | 0 |
| 489 | *Eisenbergiella massiliensis* | 0 | 490 | *Eisenbergiella tayi* | 0 |
| 491 | *Enorma timonensis* | 0 | 492 | *Enterobacter cloacae complex* | 0 |
| 493 | *Enterobacter hormaechei* | 0 | 494 | *Enterobacter sp.* | 0 |
| 495 | *Enterobacter sp. 6* | 0 | 496 | *Enterobacter sp. NRRU-N20* | 0 |
| 497 | *Enterobacteriaceae bacterium 167E* | 0 | 498 | *Enterobacteriaceae bacterium Gb8* | 0 |
| 499 | *Enterococcus asini* | 0 | 500 | *Enterococcus casseliflavus* | 0 |
| 501 | *Enterococcus durans* | 0 | 502 | *Enterococcus faecalis* | 0 |
| 503 | *Enterococcus faecium* | 0 | 504 | *Enterococcus gallinarum* | 0 |
| 505 | *Enterococcus hirae* | 0 | 506 | *Enterococcus italicus* | 0 |
| 507 | *Enterococcus sp. icri5* | 0 | 508 | *Enterorhabdus sp. Marseille-P3203T* | 0 |
| 509 | *Erwinia teleogrylli* | 0 | 510 | *Erysipelatoclostridium ramosum* | 0 |
| 511 | *Erysipelothrix larvae* | 0 | 512 | *Erysipelotrichaceae bacterium 6 1 45* | 0 |
| 513 | *Erysipelotrichaceae bacterium canine oral taxon 255* | 0 | 514 | *Erysipelotrichaceae bacterium canine oral taxon 302* | 0 |
| 515 | *Erysipelotrichaceae bacterium canine oral taxon 381* | 0 | 516 | *Erythrobacter luteus* | 0 |
| 517 | *Escherichia albertii* | 0 | 518 | *Escherichia sp.* | 0 |
| 519 | *Eubacteriaceae bacterium CHKCI004* | 0 | 520 | *Eubacteriaceae bacterium CHKCI005* | 0 |
| 521 | *Eubacterium coprostanoligenes* | 0 | 522 | *Eubacterium hallii* | 0 |
| 523 | *Eubacterium plexicaudatum* | 0 | 524 | *Eubacterium ramulus* | 0 |
| 525 | *Eubacterium rangiferina* | 0 | 526 | *Eubacterium ruminantium* | 0 |
| 527 | *Eubacterium sp. oral clone BU014* | 0 | 528 | *Eubacterium sp. oral clone EI074* | 0 |
| 529 | *Eubacterium sp CAG 180* | 0 | 530 | *Eubacterium sp CAG 274* | 0 |
| 531 | *Eubacterium uniforme* | 0 | 532 | *Eubacterium ventriosum* | 0 |
| 533 | *Extibacter muris 0.0* | 0 | 534 | *Faecalitalea cylindroides* | 0 |
| 535 | *Falcatimonas natans* | 0 | 536 | *Fastidiosipila sanguinis* | 0 |
| 537 | *Fenollaria timonensis* | 0 | 538 | *Filimonas sp. R5-375* | 0 |
| 539 | *Firmicutes bacterium* | 0 | 540 | *Firmicutes bacterium CAG345* | 0 |
| 541 | *Firmicutes bacterium CAG822* | 0 | 542 | *Firmicutes bacterium CAG 110* | 0 |
| 543 | *Firmicutes bacterium CAG 194 44 15* | 0 | 544 | *Firmicutes bacterium CAG 646* | 0 |
| 545 | *Firmicutes bacterium CAG 94* | 0 | 546 | *Firmicutes bacterium DJF VP44* | 0 |
| 547 | *Firmicutes bacterium ZOR0006* | 0 | 548 | *Firmicutes bacterium canine oral taxon 309* | 0 |
| 549 | *Flaviaesturariibacter sp.* | 0 | 550 | *Flavobacteriales endosymbiont of Carulaspis juniperi D241* | 0 |
| 551 | *Flavobacterium caeni* | 0 | 552 | *Flavobacterium sp. enrichment culture clone SA NR2* | 0 |
| 553 | *Frankiales bacterium* | 0 | 554 | *Frederiksenia canicola* | 0 |
| 555 | *Fusobacteriaceae bacterium F3 0.794000000000096* | 0 | 556 | *Fusobacterium equinum* | 0 |
| 557 | *Fusobacterium necrophorum* | 0 | 558 | *Fusobacterium nucleatum* | 0 |
| 559 | *Fusobacterium periodonticum* | 0 | 560 | *Fusobacterium russii 1.0* | 0 |
| 561 | *Fusobacterium sp. PH5-29* | 0 | 562 | *Fusobacterium ulcerans* | 0 |
| 563 | *Gemmatimonas sp. enrichment culture clone AOCRB-EC-6* | 0 | 564 | *Gemmatirosa kalamazoonesis* | 0 |
| 565 | *Globicatella sp. canine oral taxon 218* | 0 | 566 | *Gorbachella massiliensis* | 0 |
| 567 | *Gordonibacter urolithinfaciens* | 0 | 568 | *Gossypium arboreum* | 0 |
| 569 | *Gracilibacter thermotolerans* | 0 | 570 | *Gracilibacteria bacterium canine oral taxon 291* | 0 |
| 571 | *Gracilibacteria bacterium canine oral taxon 323* | 0 | 572 | *Gracilibacteria bacterium canine oral taxon 394* | 0 |
| 573 | *Granulicatella sp. canine oral taxon 095* | 0 | 574 | *Granulicella sp. 5B5* | 0 |
| 575 | *Granulicella tundricola* | 0 | 576 | *Haemophilus haemolyticus* | 0 |
| 577 | *Haemophilus massiliensis* | 0 | 578 | *Haemophilus parainfluenzae* | 0 |
| 579 | *Haemophilus pittmaniae* | 0 | 580 | *Haemophilus sp HMSC71H05* | 0 |
| 581 | *Haemophilus sputorum* | 0 | 582 | *Halanaerobaculum tunisiense* | 0 |
| 583 | *Halotalea alkalilenta* | 0 | 584 | *Helianthus annuus (common sunflower)* | 0 |
| 585 | *Helicobacter pylori* | 0 | 586 | *Helicobacter sp. LNB2F* | 0 |
| 587 | *Hespellia porcina* | 0 | 588 | *Holdemanella biformis* | 0 |
| 589 | *Holdemania filiformis* | 0 | 590 | *Holophaga sp. WY42* | 0 |
| 591 | *Howardella ureilytica* | 0 | 592 | *Hungatella hathewayi* | 0 |
| 593 | *Hydra vulgaris* | 0 | 594 | *Hydrocarboniphaga daqingensis* | 0 |
| 595 | *Hymenobacter perfusus* | 0 | 596 | *Hymenobacter sp. DG11A* | 0 |
| 597 | *Idiomarina sp.* | 0 | 598 | *Ihubacter massiliensis* | 0 |
| 599 | *Ileibacterium massiliense* | 0 | 600 | *Intestinibacillus massiliensis* | 0 |
| 601 | *Intestinibacter bartlettii* | 0 | 602 | *Intestinimonas butyriciproducens* | 0 |
| 603 | *Intestinimonas timonensis* | 0 | 604 | *Irregularibacter muris* | 0 |
| 605 | *Janthinobacterium sp. SON-1402* | 0 | 606 | *Jatrophihabitans sp.* | 0 |
| 607 | *Johnsonella ignava* | 0 | 608 | *Klebsiella michiganensis* | 0 |
| 609 | *Klebsiella oxytoca* | 0 | 610 | *Klebsiella pneumoniae* | 0 |
| 611 | *Klebsiella quasipneumoniae* | 0 | 612 | *Klebsiella variicola* | 0 |
| 613 | *Kuruna debilis 0.63900000000001* | 0 | 614 | *Lachnoclostridium phocaeense 1.0* | 0 |
| 615 | *Lachnoclostridium phytofermentans* | 0 | 616 | *Lachnoclostridium sp An181* | 0 |
| 617 | *Lachnospira pectinoschiza* | 0 | 618 | *Lachnospiraceae bacterium* | 0 |
| 619 | *Lachnospiraceae bacterium 19gly4* | 0 | 620 | *Lachnospiraceae bacterium 1 4 56FAA* | 0 |
| 621 | *Lachnospiraceae bacterium 607* | 0 | 622 | *Lachnospiraceae bacterium BTY6* | 0 |
| 623 | *Lachnospiraceae bacterium DJF CP76* | 0 | 624 | *Lachnospiraceae bacterium DJF VR44* | 0 |
| 625 | *Lachnospiraceae bacterium DW22* | 0 | 626 | *Lachnospiraceae bacterium E7* | 0 |
| 627 | *Lachnospiraceae bacterium RM29* | 0 | 628 | *Lachnospiraceae bacterium XBB1006* | 0 |
| 629 | *Lachnospiraceae bacterium canine oral taxon 037* | 0 | 630 | *Lachnospiraceae bacterium canine oral taxon 099* | 0 |
| 631 | *Lachnospiraceae bacterium canine oral taxon 156* | 0 | 632 | *Lachnospiraceae bacterium canine oral taxon 346 0.0* | 0 |
| 633 | *Lachnospiraceae bacterium canine oral taxon 399* | 0 | 634 | *Lachnospiraceae bacterium oral taxon F15* | 0 |
| 635 | *Lachnospiraceae oral clone MCE10 236* | 0 | 636 | *Lacticigenium naphtae* | 0 |
| 637 | *Lactobacillus acidophilus* | 0 | 638 | *Lactobacillus casei* | 0 |
| 639 | *Lactobacillus coleohominis* | 0 | 640 | *Lactobacillus crispatus* | 0 |
| 641 | *Lactobacillus delbrueckii* | 0 | 642 | *Lactobacillus fermentum* | 0 |
| 643 | *Lactobacillus gasseri* | 0 | 644 | *Lactobacillus kalixensis* | 0 |
| 645 | *Lactobacillus kunkeei 1.0* | 0 | 646 | *Lactobacillus pantheris* | 0 |
| 647 | *Lactobacillus paragasseri* | 0 | 648 | *Lactobacillus plantarum* | 0 |
| 649 | *Lactobacillus pontis* | 0 | 650 | *Lactobacillus reuteri* | 0 |
| 651 | *Lactobacillus ruminis* | 0 | 652 | *Lactobacillus salivarius* | 0 |
| 653 | *Lactobacillus sanfranciscensis* | 0 | 654 | *Lactobacillus sp. C30An8* | 0 |
| 655 | *Lactobacillus sp. KC45b* | 0 | 656 | *Lactobacillus sp. QAULB01* | 0 |
| 657 | *Lactococcus lactis* | 0 | 658 | *Lautropia mirabilis* | 0 |
| 659 | *Lawsonella clevelandensis* | 0 | 660 | *Lawsonia intracellularis* | 0 |
| 661 | *Lawsonibacter asaccharolyticus* | 0 | 662 | *Leadbetterella byssophila* | 0 |
| 663 | *Legionella geestiana* | 0 | 664 | *Leptolyngbya sp. WJT66-NPBG2* | 0 |
| 665 | *Leptotrichia buccalis* | 0 | 666 | *Leptotrichia goodfellowii* | 0 |
| 667 | *Leptotrichia shahii* | 0 | 668 | *Leptotrichia sp. ES2714 GLU* | 0 |
| 669 | *Leptotrichia sp. oral taxon 212* | 0 | 670 | *Leucobacter sp. KUCd3* | 0 |
| 671 | *Libanicoccus massiliensis* | 0 | 672 | *Listeria monocytogenes* | 0 |
| 673 | *Loktanella sp. NP29* | 0 | 674 | *Longimicrobium terrae* | 0 |
| 675 | *Luedemannella sp. 119-1-07* | 0 | 676 | *Luteolibacter algae* | 0 |
| 677 | *Macrochaete psychrophila* | 0 | 678 | *Mailhella massiliensis* | 0 |
| 679 | *Marseilla massiliensis* | 0 | 680 | *Marseillibacter massiliensis* | 0 |
| 681 | *Massilimaliae massiliensis* | 0 | 682 | *Massilioclostridium coli* | 0 |
| 683 | *Massiliprevotella massiliensis* | 0 | 684 | *Mediterranea massiliensis* | 0 |
| 685 | *Megamonas funiformis CAG 377* | 0 | 686 | *Megamonas hypermegale* | 0 |
| 687 | *Megasphaera cerevisiae* | 0 | 688 | *Megasphaera micronuciformis* | 0 |
| 689 | *Megasphaera sp. DISK 18* | 0 | 690 | *Megasphaera sp. oral clone BS073* | 0 |
| 691 | *Megasphaera sp. oral clone CS025* | 0 | 692 | *Melghirimyces thermohalophilus* | 0 |
| 693 | *Merdibacter massiliensis* | 0 | 694 | *Mesocricetibacter intestinalis* | 0 |
| 695 | *Methanobrevibacter smithii* | 0 | 696 | *Methanobrevibacter sp.* | 0 |
| 697 | *Methanobrevibacter sp. R4C* | 0 | 698 | *Methanosphaera stadtmanae* | 0 |
| 699 | *Methylobacterium variabile* | 0 | 700 | *Microcystis sp. SAG 43.90* | 0 |
| 701 | *Mobilicoccus sp. YIM 101593* | 0 | 702 | *Mobilisporobacter senegalensis* | 0 |
| 703 | *Monoglobus pectinilyticus* | 0 | 704 | *Moraxella bovoculi* | 0 |
| 705 | *Moraxella lincolnii* | 0 | 706 | *Moraxella osloensis* | 0 |
| 707 | *Mordavella massiliensis* | 0 | 708 | *Morganella morganii* | 0 |
| 709 | *Moryella indoligenes* | 0 | 710 | *Mucilaginibacter auburnensis* | 0 |
| 711 | *Mucilaginibacter sp. 1042* | 0 | 712 | *Murdochiella massiliensis* | 0 |
| 713 | *Muribacter muris* | 0 | 714 | *Murimonas intestini* | 0 |
| 715 | *Mycobacterium tuberculosis* | 0 | 716 | *Mycoplasma fastidiosum* | 0 |
| 717 | *Mycoplasma feliminutum* | 0 | 718 | *Mycoplasma molare* | 0 |
| 719 | *Myroides marinus* | 0 | 720 | *Myxococcales bacterium LWH177* | 0 |
| 721 | *Nannocystis sp.* | 0 | 722 | *Natranaerovirga pectinivora* | 0 |
| 723 | *Natronincola histidinovorans* | 0 | 724 | *Negativicoccus massiliensis 0.0* | 0 |
| 725 | *Neisseria meningitidis* | 0 | 726 | *Neisseria sp. TID-16* | 0 |
| 727 | *Neisseria zoodegmatis* | 0 | 728 | *Niabella ginsengisoli* | 0 |
| 729 | *Niameybacter massiliensis* | 0 | 730 | *Nicotiana otophora* | 0 |
| 731 | *Nitriliruptor alkaliphilus* | 0 | 732 | *Nitrospira japonica* | 0 |
| 733 | *Nocardioides sp.* | 0 | 734 | *Octadecabacter temperatus* | 0 |
| 735 | *Odoribacter laneus* | 0 | 736 | *Olivibacter ginsengisoli* | 0 |
| 737 | *Olsenella genomosp. C1* | 0 | 738 | *Olsenella sp. D6* | 0 |
| 739 | *Olsenella sp. SIT9* | 0 | 740 | *Opitutae bacterium SCGC AG-212-L18* | 0 |
| 741 | *Oryza meyeriana* | 0 | 742 | *Oryza sativa Indica Group (long-grained rice)* | 0 |
| 743 | *Oscillibacter sp. ER4* | 0 | 744 | *Oscillibacter sp. G2* | 0 |
| 745 | *Oscillibacter sp. Marseille-P3302* | 0 | 746 | *Oscillibacter sp 57 20* | 0 |
| 747 | *Oscillibacter sp CAG 241* | 0 | 748 | *Oscillospira guilliermondii* | 0 |
| 749 | *Oscillospiraceae bacterium Zagget4* | 0 | 750 | *Ottowia sp. oral taxon 894* | 0 |
| 751 | *Oxalobacter formigenes* | 0 | 752 | *Paenibacillus taiwanensis 0.0* | 0 |
| 753 | *Pantoea stewartii 0.0* | 0 | 754 | *Papillibacter cinnamivorans* | 0 |
| 755 | *Parabacteroides chinchillae* | 0 | 756 | *Parabacteroides johnsonii* | 0 |
| 757 | *Parabacteroides sp.* | 0 | 758 | *Parabacteroides sp. Marseille-P3668* | 0 |
| 759 | *Parabacteroides sp. PH5-33* | 0 | 760 | *Parabacteroides sp. YL27* | 0 |
| 761 | *Parabacteroides sp CAG 409* | 0 | 762 | *Paraclostridium bifermentans 1.84471205263304* | 0 |
| 763 | *Paracraurococcus ruber* | 0 | 764 | *Paracraurococcus sp. 1PNM-27* | 0 |
| 765 | *Paraphelidium tribonemae* | 0 | 766 | *Paraprevotella xylaniphila* | 0 |
| 767 | *Parasporobacterium paucivorans* | 0 | 768 | *Parasutterella secunda* | 0 |
| 769 | *Parvimonas micra* | 0 | 770 | *Parviterribacter multiflagellatus* | 0 |
| 771 | *Pasteurella multocida* | 0 | 772 | *Pasteurella sp. C9I9* | 0 |
| 773 | *Pasteurellaceae bacterium canine oral taxon 271* | 0 | 774 | *Pasteurellaceae bacterium canine oral taxon 272* | 0 |
| 775 | *Patulibacter sp. S1-28* | 0 | 776 | *Pediococcus acidilactici* | 0 |
| 777 | *Pediococcus pentosaceus* | 0 | 778 | *Pediococcus sp. T1R4C24* | 0 |
| 779 | *Peptococcus simiae* | 0 | 780 | *Peptoniphilus catoniae* | 0 |
| 781 | *Peptoniphilus sp. Dr06* | 0 | 782 | *Peptoniphilus tyrrelliae* | 0 |
| 783 | *Peptostreptococcaceae bacterium VA2* | 0 | 784 | *Peptostreptococcaceae bacterium canine oral taxon 065* | 0 |
| 785 | *Peptostreptococcaceae bacterium canine oral taxon 124* | 0 | 786 | *Peptostreptococcaceae bacterium canine oral taxon 155* | 0 |
| 787 | *Peptostreptococcus anaerobius* | 0 | 788 | *Persicaria minor* | 0 |
| 789 | *Phascolarctobacterium faecium* | 0 | 790 | *Phascolarctobacterium sp. 377* | 0 |
| 791 | *Phascolarctobacterium sp. canine oral taxon 212* | 0 | 792 | *Phascolarctobacterium sp CAG 266* | 0 |
| 793 | *Phascolarctobacterium succinatutens* | 0 | 794 | *Phaselicystis flava* | 0 |
| 795 | *Phreatobacter sp. S-12* | 0 | 796 | *Planctomyces sp.* | 0 |
| 797 | *Planctomycetaceae bacterium LX124* | 0 | 798 | *Planctomycetales bacterium Ellin6207* | 0 |
| 799 | *Planococcaceae bacterium Storch 2/2-2* | 0 | 800 | *Polymorphobacter sp.* | 0 |
| 801 | *Porphyromonadaceae bacterium CG2 30 38 12* | 0 | 802 | *Porphyromonas asaccharolytica* | 0 |
| 803 | *Porphyromonas endodontalis* | 0 | 804 | *Porphyromonas sp. HMSC065F10* | 0 |
| 805 | *Porphyromonas sp. canine oral taxon 181* | 0 | 806 | *Porphyromonas sp. canine oral taxon 366* | 0 |
| 807 | *Porphyromonas sp. canine oral taxon 401* | 0 | 808 | *Porphyromonas sp. feline oral taxon 110* | 0 |
| 809 | *Porphyromonas uenonis* | 0 | 810 | *Prevotella bivia* | 0 |
| 811 | *Prevotella buccae* | 0 | 812 | *Prevotella copri* | 0 |
| 813 | *Prevotella corporis* | 0 | 814 | *Prevotella denticola* | 0 |
| 815 | *Prevotella disiens* | 0 | 816 | *Prevotella enoeca 0.0* | 0 |
| 817 | *Prevotella intermedia* | 0 | 818 | *Prevotella jejuni 0.0090597172079156* | 0 |
| 819 | *Prevotella micans* | 0 | 820 | *Prevotella nanceiensis 0.0* | 0 |
| 821 | *Prevotella nigrescens* | 0 | 822 | *Prevotella oris* | 0 |
| 823 | *Prevotella sp. 109* | 0 | 824 | *Prevotella sp. AN 5135* | 0 |
| 825 | *Prevotella sp. P2A FAAD4 0.0* | 0 | 826 | *Prevotella sp. canine oral taxon 282* | 0 |
| 827 | *Prevotella sp. canine oral taxon 372* | 0 | 828 | *Prevotella sp. oral clone ASCG12 1.58496250072116* | 0 |
| 829 | *Prevotella sp. oral clone ID019* | 0 | 830 | *Prevotella sp AM42 24* | 0 |
| 831 | *Prevotella sp CAG 1185* | 0 | 832 | *Prevotella sp CAG 279* | 0 |
| 833 | *Prevotella sp CAG 891* | 0 | 834 | *Prevotella stercorea* | 0 |
| 835 | *Prevotella timonensis* | 0 | 836 | *Prevotella veroralis 1.58496250072116* | 0 |
| 837 | *Prevotellamassilia timonensis* | 0 | 838 | *Proteus hauseri* | 0 |
| 839 | *Proteus mirabilis* | 0 | 840 | *Proteus penneri* | 0 |
| 841 | *Pseudanabaena galeata* | 0 | 842 | *Pseudobutyrivibrio sp. CA38* | 0 |
| 843 | *Pseudoflavitalea soli* | 0 | 844 | *Pseudoflavonifractor capillosus* | 0 |
| 845 | *Pseudoflavonifractor sp. Marseille-P3106* | 0 | 846 | *Pseudoflavonifractor sp An184* | 0 |
| 847 | *Pseudomonas aeruginosa group* | 0 | 848 | *Pseudomonas brassicacearum* | 0 |
| 849 | *Pseudomonas fluorescens* | 0 | 850 | *Pseudoxanthomonas mexicana 0.0003926228588268* | 0 |
| 851 | *Pygmaiobacter massiliensis* | 0 | 852 | *Raoultibacter timonensis* | 0 |
| 853 | *Rappaport israeli* | 0 | 854 | *Rarimicrobium hominis* | 0 |
| 855 | *Rhodobacter sp. JA352* | 0 | 856 | *Rhodocista sp. SCSIO 13435* | 0 |
| 857 | *Rhodocytophaga aerolata* | 0 | 858 | *Rhodopila globiformis* | 0 |
| 859 | *Rhodoplanes sp. enrichment culture clone ANA-RAS-62* | 0 | 860 | *Rhodopseudomonas sp. CZ-1* | 0 |
| 861 | *Riemerella columbina* | 0 | 862 | *Rodentibacter pneumotropicus* | 0 |
| 863 | *Romboutsia sp. MT17* | 0 | 864 | *Roseburia intestinalis* | 0 |
| 865 | *Roseburia sp. 1120* | 0 | 866 | *Roseburia sp. 499* | 0 |
| 867 | *Roseburia sp. 831b* | 0 | 868 | *Roseburia sp CAG 303* | 0 |
| 869 | *Roseomonas frigidaquae* | 0 | 870 | *Roseomonas sp.* | 0 |
| 871 | *Roseomonas sp. Atb2* | 0 | 872 | *Roseomonas sp. L1B40* | 0 |
| 873 | *Roseomonas sp. P-9Y* | 0 | 874 | *Roseomonas sp. S2-87* | 0 |
| 875 | *Roseomonas sp. ZSGR23* | 0 | 876 | *Rothia dentocariosa* | 0 |
| 877 | *Rothia sp. HMSC065C03* | 0 | 878 | *Rubellimicrobium mesophilum* | 0 |
| 879 | *Rubritepida sp. MDT2-1-1* | 0 | 880 | *Rubrobacter radiotolerans* | 0 |
| 881 | *Ruminococcaceae bacterium* | 0 | 882 | *Ruminococcaceae bacterium D5* | 0 |
| 883 | *Ruminococcaceae bacterium Marseille-P2935* | 0 | 884 | *Ruminococcaceae bacterium Marseille-P3449* | 0 |
| 885 | *Ruminococcaceae bacterium Marseille-P3738* | 0 | 886 | *Ruminococcaceae bacterium cv2* | 0 |
| 887 | *Ruminococcus bicirculans* | 0 | 888 | *Ruminococcus champanellensis* | 0 |
| 889 | *Ruminococcus faecis* | 0 | 890 | *Ruminococcus flavefaciens* | 0 |
| 891 | *Ruminococcus gauvreauii* | 0 | 892 | *Ruminococcus sp. 14531* | 0 |
| 893 | *Ruminococcus sp. 653* | 0 | 894 | *Ruminococcus sp. AT10* | 0 |
| 895 | *Ruminococcus sp. CE2* | 0 | 896 | *Ruminococcus sp. DJF VR70k1* | 0 |
| 897 | *Ruminococcus sp. ID1* | 0 | 898 | *Ruminococcus sp. NK3A76* | 0 |
| 899 | *Ruminococcus sp. Pei041* | 0 | 900 | *Ruminococcus sp. RLB3* | 0 |
| 901 | *Ruminococcus sp. W22* | 0 | 902 | *Ruminococcus sp. WAL 17306* | 0 |
| 903 | *Ruminococcus sp. YE281* | 0 | 904 | *Ruminococcus sp. YE58* | 0 |
| 905 | *SR1 bacterium canine oral taxon 382* | 0 | 906 | *Saccharopolyspora rectivirgula* | 0 |
| 907 | *Salinirepens amamiensis* | 0 | 908 | *Sediminibacterium sp. LT21-MRL* | 0 |
| 909 | *Segetibacter aerophilus* | 0 | 910 | *Selenomonas sp. canine oral taxon 167* | 0 |
| 911 | *Sellimonas intestinalis* | 0 | 912 | *Serratia marcescens* | 0 |
| 913 | *Serratia symbiotica* | 0 | 914 | *Shigella dysenteriae* | 0 |
| 915 | *Shigella sonnei* | 0 | 916 | *Simonsiella muelleri* | 0 |
| 917 | *Solanum lycopersicum (tomato)* | 0 | 918 | *Solimonas sp. CDMK* | 0 |
| 919 | *Sorangiineae bacterium 706* | 0 | 920 | *Sorangium cellulosum* | 0 |
| 921 | *Sphingobacterium sp. GR16* | 0 | 922 | *Sphingobacterium sp. P-7* | 0 |
| 923 | *Sphingobacterium sp. YK2* | 0 | 924 | *Sphingomonas metalli* | 0 |
| 925 | *Sphingomonas sp.* | 0 | 926 | *Sphingomonas sp. ID1715* | 0 |
| 927 | *Sphingomonas sp. W2.10-27* | 0 | 928 | *Sphingomonas vulcanisoli* | 0 |
| 929 | *Spirochaeta sp. canine oral taxon 314* | 0 | 930 | *Spirochaetales bacterium Spiro 06* | 0 |
| 931 | *Spongiibacter sp. CC-AMW-B* | 0 | 932 | *Sporocytophaga sp. 4v* | 0 |
| 933 | *Sporomusaceae bacterium MMFC1* | 0 | 934 | *Staphylococcus epidermidis* | 0 |
| 935 | *Staphylococcus sp. Rai11 0.0* | 0 | 936 | *Starria zimbabweensis* | 0 |
| 937 | *Stenotrophomonas sp. 2012A* | 0 | 938 | *Streptococcus agalactiae* | 0 |
| 939 | *Streptococcus alactolyticus* | 0 | 940 | *Streptococcus canis* | 0 |
| 941 | *Streptococcus equi* | 0 | 942 | *Streptococcus equinus* | 0 |
| 943 | *Streptococcus ferus* | 0 | 944 | *Streptococcus hongkongensis* | 0 |
| 945 | *Streptococcus intermedius 1.0* | 0 | 946 | *Streptococcus lutetiensis* | 0 |
| 947 | *Streptococcus pneumoniae* | 0 | 948 | *Streptococcus porci 0.63900000000001* | 0 |
| 949 | *Streptococcus sp.* | 0 | 950 | *Streptococcus sp. oral clone ASCB12* | 0 |
| 951 | *Streptococcus sp. oral clone ASCC01* | 0 | 952 | *Streptococcus sp. oral taxon G59* | 0 |
| 953 | *Streptococcus suis* | 0 | 954 | *Streptococcus thermophilus* | 0 |
| 955 | *Sutterella parvirubra* | 0 | 956 | *Sutterella sp. 252* | 0 |
| 957 | *Sutterella sp. Marseille-P3660* | 0 | 958 | *Sutterella sp. YIT 12072* | 0 |
| 959 | *Sutterella wadsworthensis* | 0 | 960 | *Syntrophococcus sucromutans* | 0 |
| 961 | *Terrimonas sp.* | 0 | 962 | *Terrimonas sp. 16-45A* | 0 |
| 963 | *Terrisporobacter glycolicus* | 0 | 964 | *Thermasporomyces composti* | 0 |
| 965 | *Thermicanus aegyptius* | 0 | 966 | *Traorella massiliensis* | 0 |
| 967 | *Treponema sp. canine oral taxon 199* | 0 | 968 | *Treponema sp. canine oral taxon 246* | 0 |
| 969 | *Treponema sp. feline oral taxon 123* | 0 | 970 | *Treponema sp. feline oral taxon 209* | 0 |
| 971 | *Trichinella pseudospiralis* | 0 | 972 | *Trichopus sempervirens* | 0 |
| 973 | *Trimenia moorei* | 0 | 974 | *Triticum aestivum (bread wheat)* | 0 |
| 975 | *Truepera radiovictrix* | 0 | 976 | *Turicibacter sanguinis* | 0 |
| 977 | *Turicimonas muris* | 0 | 978 | *Tyzzerella nexilis* | 0 |
| 979 | *Tyzzerella sp. Marseille-P3062* | 0 | 980 | *Ureaplasma canigenitalium* | 0 |
| 981 | *Variibacter gotjawalensis* | 0 | 982 | *Veillonella caviae* | 0 |
| 983 | *Veillonella dispar* | 0 | 984 | *Veillonella infantium* | 0 |
| 985 | *Veillonella parvula* | 0 | 986 | *Veillonella ratti* | 0 |
| 987 | *Veillonella seminalis* | 0 | 988 | *Veillonella sp. oral clone OH1A* | 0 |
| 989 | *Veillonella sp. oral taxon 780* | 0 | 990 | *Veillonella sp CAG 933* | 0 |
| 991 | *Veillonella sp T11011* | 0 | 992 | *Veillonellaceae bacterium DNF00626* | 0 |
| 993 | *Veillonellaceae bacterium KA00182* | 0 | 994 | *Veillonellaceae bacterium canine oral taxon 211* | 0 |
| 995 | *Verrucomicrobia bacterium OR-59* | 0 | 996 | *Verrucomicrobia bacterium WY51* | 0 |
| 997 | *Verrucomicrobiaceae bacterium SCGC AG-212-N21* | 0 | 998 | *Wodyetia bifurcata* | 0 |
| 999 | *Xenorhabdus stockiae* | 0 | 1000 | *Xylochloris irregularis* | 0 |

**Supplementary Table 2** The SHAP value of each specie in 10-species signature-based IBD classification model.

| The microbial species | SHAP values |
| --- | --- |
| *Dorea formicigenerans* | 0.8558375 |
| *Oscilibacter massiliensis* | 0.59565663 |
| *Anaerotruncus rubiinfantis* | 0.5003931 |
| *Fusicatenibacter saccharivorans* | 0.47928274 |
| *Collinsella aerofaciens* | 0.36147773 |
| *Ruminococcus bromii* | 0.28632367 |
| *Akkermansia muciniphila* | 0.25235325 |
| *Alistipes finegoldii* | 0.2488213 |
| *Clostridioides difficile* | 0.23552625 |
| *Eubacterium oxidoreducens* | 0.20630014 |

**Supplementary Table 3** The SHAP value of each specie in the top 250 variable species-based UC/CD classification model.

| Number | The microbial species | SHAP values | Number | The microbial species | SHAP values |
| --- | --- | --- | --- | --- | --- |
| 1 | *Odoribacter splanchnicus* | 0.516948 | 2 | *Bacteroides xylanisolvens* | 0.312179 |
| 3 | *Clostridium clostridioforme* | 0.284963 | 4 | *Akkermansia muciniphila* | 0.284112 |
| 5 | *Dorea formicigenerans* | 0.263913 | 6 | *Proteobacteria bacterium CAG 139* | 0.229952 |
| 7 | *Dialister invisus* | 0.211532 | 8 | *Alistipes putredinis* | 0.177872 |
| 9 | *Veillonella parvula* | 0.145024 | 10 | *Eubacterium hallii* | 0.140922 |
| 11 | *Bifidobacterium adolescentis* | 0.135427 | 12 | *Roseburia intestinalis* | 0.107644 |
| 13 | *Bifidobacterium longum* | 0.105674 | 14 | *Bacteroides eggerthii* | 0.092923 |
| 15 | *Dialister sp CAG 357* | 0.09208 | 16 | *Roseburia inulinivorans* | 0.091828 |
| 17 | *Clostridium bolteae* | 0.09004 | 18 | *Bacteroides massiliensis* | 0.079449 |
| 19 | *Collinsella aerofaciens* | 0.067799 | 20 | *Bacteroides sp CAG 144* | 0.066866 |
| 21 | *Bacteroides thetaiotaomicron* | 0.065678 | 22 | *Intestinimonas butyriciproducens* | 0.065295 |
| 23 | *Bacteroides finegoldii* | 0.063873 | 24 | *Methanobrevibacter smithii* | 0.062397 |
| 25 | *Paraprevotella clara* | 0.0605 | 26 | *Blautia obeum* | 0.060499 |
| 27 | *Eubacterium eligens* | 0.058999 | 28 | *Lachnospira pectinoschiza* | 0.058966 |
| 29 | *Bacteroides cellulosilyticus* | 0.056009 | 30 | *Bacteroides faecis* | 0.055989 |
| 31 | *Enterococcus faecium* | 0.055763 | 32 | *Anaerotruncus sp CAG 528* | 0.054225 |
| 33 | *Firmicutes bacterium CAG 83* | 0.047502 | 34 | *Bacteroides fluxus* | 0.045951 |
| 35 | *Ruminococcus lactaris* | 0.043612 | 36 | *Eubacterium sp CAG 251* | 0.041845 |
| 37 | *Parasutterella excrementihominis* | 0.037592 | 38 | *Veillonella dispar* | 0.03713 |
| 39 | *Barnesiella intestinihominis* | 0.036375 | 40 | *Streptococcus parasanguinis* | 0.035405 |
| 41 | *Bacteroides stercoris* | 0.03384 | 42 | *Eisenbergiella massiliensis* | 0.033728 |
| 43 | *Alistipes finegoldii* | 0.033003 | 44 | *Ruminococcus gnavus* | 0.031903 |
| 45 | *Bifidobacterium animalis* | 0.03071 | 46 | *Eubacterium rectale* | 0.028852 |
| 47 | *Bifidobacterium bifidum* | 0.026666 | 48 | *Parabacteroides merdae* | 0.026346 |
| 49 | *Lactobacillus acidophilus* | 0.025211 | 50 | *Eubacterium sp CAG 38* | 0.02383 |
| 51 | *Prevotella sp CAG 1185* | 0.023356 | 52 | *Bacteroides salyersiae* | 0.023313 |
| 53 | *Clostridium symbiosum* | 0.020115 | 54 | *Agathobaculum butyriciproducens* | 0.019368 |
| 55 | *Bacteroides fragilis* | 0.018928 | 56 | *Bifidobacterium pseudocatenulatum* | 0.018927 |
| 57 | *Eubacterium siraeum* | 0.018699 | 58 | *Roseburia faecis* | 0.017961 |
| 59 | *Bacteroides caccae* | 0.017553 | 60 | *Prevotella sp CAG 891* | 0.014946 |
| 61 | *Anaerostipes hadrus* | 0.014808 | 62 | *Bacteroides dorei* | 0.013155 |
| 63 | *Dorea longicatena* | 0.013058 | 64 | *Bacteroides ovatus* | 0.013 |
| 65 | *Clostridium leptum* | 0.012557 | 66 | *Escherichia coli* | 0.012522 |
| 67 | *Clostridium sp CAG 58* | 0.012519 | 68 | *Bacteroides vulgatus* | 0.012332 |
| 69 | *Coprobacter fastidiosus* | 0.011915 | 70 | *Faecalibacterium prausnitzii* | 0.01117 |
| 71 | *Blautia sp CAG 257* | 0.011133 | 72 | *Ruminococcus bicirculans* | 0.011025 |
| 73 | *Parabacteroides distasonis* | 0.010978 | 74 | *Alistipes shahii* | 0.010521 |
| 75 | *Bacteroides faecis CAG 32* | 0.010376 | 76 | *Eubacterium sp CAG 180* | 0.009782 |
| 77 | *Eubacterium ventriosum* | 0.00906 | 78 | *Acidaminococcus intestini* | 0.008843 |
| 79 | *Prevotella copri* | 0.008643 | 80 | *Coprococcus catus* | 0.008019 |
| 81 | *Bilophila wadsworthia* | 0.006554 | 82 | *Bacteroides uniformis* | 0.005841 |
| 83 | *Erysipelotrichaceae bacterium 6 1 45* | 0.00577 | 84 | *Erysipelatoclostridium ramosum* | 0.005333 |
| 85 | *Streptococcus salivarius* | 0.005292 | 86 | *Haemophilus parainfluenzae* | 0.005178 |
| 87 | *Hungatella hathewayi* | 0.005141 | 88 | *Ruminococcus bromii* | 0.004442 |
| 89 | *Ruminococcus torques* | 0.004151 | 90 | *Clostridium saccharolyticum* | 0.003941 |
| 91 | *Coprococcus comes* | 0.003751 | 92 | *Phascolarctobacterium faecium* | 0.003648 |
| 93 | *Ruthenibacterium lactatiformans* | 0.003411 | 94 | *Clostridium bolteae CAG 59* | 0.002824 |
| 95 | *Fusobacterium nucleatum* | 0.001557 | 96 | *Aggregatibacter actinomycetemcomitans* | 0 |
| 97 | *Aggregatibacter aphrophilus 0* | 0 | 98 | *Alistipes onderdonkii* | 0 |
| 99 | *Alistipes sp. AL-1* | 0 | 100 | *Alistipes sp. RMA 9912* | 0 |
| 101 | *Alkaliphilus sp. LacT* | 0 | 102 | *Alloprevotella tannerae* | 0 |
| 103 | *Anaerococcus sp. S138* | 0 | 104 | *Anaerotruncus rubiinfantis* | 0 |
| 105 | *Angelakisella massiliensis* | 0 | 106 | *Bacteroides clarus* | 0 |
| 107 | *Bacteroides coprocola* | 0 | 108 | *Bacteroides gallinaceum* | 0 |
| 109 | *Bacteroides intestinalis* | 0 | 110 | *Bacteroides plebeius* | 0 |
| 111 | *Bacteroides sartorii* | 0 | 112 | *Bacteroides sp.* | 0 |
| 113 | *Bacteroides sp. S427* | 0 | 114 | *Bacteroides sp 43 108* | 0 |
| 115 | *Bifidobacterium aerophilum* | 0 | 116 | *Bifidobacterium dentium* | 0 |
| 117 | *Bifidobacterium reuteri* | 0 | 118 | *Bisgaard Taxon 7* | 0 |
| 119 | *Blautia hansenii* | 0 | 120 | *Blautia hydrogenotrophica* | 0 |
| 121 | *Blautia massiliensis* | 0 | 122 | *Blautia stercoris* | 0 |
| 123 | *Blautia wexlerae* | 0 | 124 | *Butyricicoccus faecihominis* | 0 |
| 125 | *Butyrivibrio sp CAG 318* | 0 | 126 | *Campylobacter concisus* | 0 |
| 127 | *Christensenella massiliensis* | 0 | 128 | *Cloacibacillus evryensis* | 0 |
| 129 | *Clostridia bacterium UC5.1-2D9* | 0 | 130 | *Clostridiaceae bacterium DJF VR09* | 0 |
| 131 | *Clostridiales bacterium 40-4c* | 0 | 132 | *Clostridiales bacterium CHKCI006* | 0 |
| 133 | *Clostridiales bacterium Firm 08* | 0 | 134 | *Clostridiales bacterium Marseille-P2846* | 0 |
| 135 | *Clostridiales bacterium NK3B98* | 0 | 136 | *Clostridioides difficile* | 0 |
| 137 | *Clostridium butyricum* | 0 | 138 | *Clostridium chauvoei* | 0 |
| 139 | *Clostridium innocuum* | 0 | 140 | *Clostridium neonatale* | 0 |
| 141 | *Clostridium septicum* | 0 | 142 | *Clostridium sp. AT4* | 0 |
| 143 | *Clostridium sp. Marseille-P2415* | 0 | 144 | *Clostridium sp. Marseille-P3244* | 0 |
| 145 | *Clostridium sp. ND2* | 0 | 146 | *Clostridium sp. T2* | 0 |
| 147 | *Collinsella bouchesdurhonensis* | 0 | 148 | *Collinsella ihuae* | 0 |
| 149 | *Collinsella phocaeensis* | 0 | 150 | *Collinsella tanakaei* | 0 |
| 151 | *Coprococcus eutactus* | 0 | 152 | *Cuneatibacter caecimuris* | 0 |
| 153 | *Dakarella massiliensis* | 0 | 154 | *Dialister pneumosintes* | 0 |
| 155 | *Dialister propionicifaciens* | 0 | 156 | *Dialister sp. S7D* | 0 |
| 157 | *Dialister sp. oral clone BS016* | 0 | 158 | *Dialister sp. oral clone FY011* | 0 |
| 159 | *Dialister sp. oral clone MCE7 134* | 0 | 160 | *Dialister succinatiphilus* | 0 |
| 161 | *Drancourtella massiliensis* | 0 | 162 | *Eggerthellaceae bacterium* | 0 |
| 163 | *Eikenella corrodens* | 0 | 164 | *Eisenbergiella tayi* | 0 |
| 165 | *Erwinia teleogrylli* | 0 | 166 | *Erysipelatoclostridium sp. SNUG30386* | 0 |
| 167 | *Escherichia albertii* | 0 | 168 | *Escherichia sp.* | 0 |
| 169 | *Eubacteriaceae bacterium CHKCI004* | 0 | 170 | *Eubacterium coprostanoligenes* | 0 |
| 171 | *Eubacterium oxidoreducens* | 0 | 172 | *Eubacterium ramulus* | 0 |
| 173 | *Fenollaria timonensis* | 0 | 174 | *Firmicutes bacterium CAG 94* | 0 |
| 175 | *Firmicutes bacterium DJF VP44* | 0 | 176 | *Firmicutes bacterium ZOR0006* | 0 |
| 177 | *Flavonifractor plautii* | 0 | 178 | *Fusicatenibacter saccharivorans* | 0 |
| 179 | *Fusobacterium equinum* | 0 | 180 | *Fusobacterium necrophorum* | 0 |
| 181 | *Haemophilus sputorum* | 0 | 182 | *Helicobacter pylori* | 0 |
| 183 | *Ileibacterium massiliense* | 0 | 184 | *Klebsiella michiganensis* | 0 |
| 185 | *Klebsiella pneumoniae* | 0 | 186 | *Klebsiella quasipneumoniae* | 0 |
| 187 | *Klebsiella variicola* | 0 | 188 | *Lachnoclostridium phytofermentans* | 0 |
| 189 | *Lachnospiraceae bacterium 1 4 56FAA* | 0 | 190 | *Lachnospiraceae bacterium BTY6* | 0 |
| 191 | *Lachnospiraceae bacterium E7* | 0 | 192 | *Lachnospiraceae bacterium XBB1006* | 0 |
| 193 | *Lachnospiraceae bacterium canine oral taxon 099* | 0 | 194 | *Lacticigenium naphtae* | 0 |
| 195 | *Lactobacillus fermentum* | 0 | 196 | *Lactobacillus salivarius* | 0 |
| 197 | *Marvinbryantia formatexigens* | 0 | 198 | *Massiliprevotella massiliensis* | 0 |
| 199 | *Megasphaera micronuciformis* | 0 | 200 | *Mordavella massiliensis* | 0 |
| 201 | *Morganella morganii* | 0 | 202 | *Negativibacillus massiliensis* | 0 |
| 203 | *Neglecta timonensis* | 0 | 204 | *Neisseria meningitidis* | 0 |
| 205 | *Niameybacter massiliensis* | 0 | 206 | *Odoribacter laneus* | 0 |
| 207 | *Oscilibacter massiliensis* | 0 | 208 | *Oscillibacter sp 57 20* | 0 |
| 209 | *Paraprevotella xylaniphila* | 0 | 210 | *Peptostreptococcaceae bacterium VA2* | 0 |
| 211 | *Phascolarctobacterium sp. 377* | 0 | 212 | *Phascolarctobacterium succinatutens* | 0 |
| 213 | *Porphyromonas sp. HMSC065F10* | 0 | 214 | *Prevotella bivia* | 0 |
| 215 | *Prevotella buccae* | 0 | 216 | *Prevotella nigrescens* | 0 |
| 217 | *Prevotella sp. 109* | 0 | 218 | *Prevotella sp. AN 5135* | 0 |
| 219 | *Prevotella sp 885* | 0 | 220 | *Prevotella sp AM42 24* | 0 |
| 221 | *Prevotella stercorea* | 0 | 222 | *Proteus mirabilis* | 0 |
| 223 | *Pseudobutyrivibrio sp. CA38* | 0 | 224 | *Pseudoflavonifractor capillosus* | 0 |
| 225 | *Raoultibacter massiliensis* | 0 | 226 | *Raoultibacter timonensis* | 0 |
| 227 | *Rarimicrobium hominis* | 0 | 228 | *Rodentibacter pneumotropicus* | 0 |
| 229 | *Romboutsia sp. MT17* | 0 | 230 | *Roseburia hominis* | 0 |
| 231 | *Rothia dentocariosa* | 0 | 232 | *Ruminococcus sp.* | 0 |
| 233 | *Ruminococcus sp. 653* | 0 | 234 | *Ruminococcus sp. AT10* | 0 |
| 235 | *Ruminococcus sp. Marseille-P328* | 0 | 236 | *Ruminococcus sp. YE281* | 0 |
| 237 | *Ruminococcus sp. YE58* | 0 | 238 | *Shigella dysenteriae* | 0 |
| 239 | *Spirochaetales bacterium Spiro 06* | 0 | 240 | *Staphylococcus epidermidis 4.32652* | 0 |
| 241 | *Streptococcus mutans* | 0 | 242 | *Streptococcus pneumoniae* | 0 |
| 243 | *Streptococcus suis* | 0 | 244 | *Sutterella wadsworthensis* | 0 |
| 245 | *Turicibacter sanguinis* | 0 | 246 | *Veillonella atypica* | 0 |
| 247 | *Veillonella magna* | 0 | 248 | *Veillonella seminalis* | 0 |
| 249 | *Veillonella sp. oral clone OH1A* | 0 | 250 | *Veillonellaceae bacterium canine oral taxon 211* | 0 |

**Supplementary Table 4** The SHAP value of each specie in 5-species signature-based UC/CD classification model.

| The microbial species | SHAP values |
| --- | --- |
| *Odoribacter splanchnicus* | 0.591921 |
| *Dorea formicigenerans* | 0.576776 |
| *Clostridium clostridioforme* | 0.488285 |
| *Bacteroides xylanisolvens* | 0.436526 |
| *Akkermansia muciniphila* | 0.354706 |

**Supplementary Table 5** The clinical data of 107 IBD patients.

| **ID** | **Diagnosis** | **Behavior** | **Location** | **Mayo score** | **CDAI** | **Type** | **Age** | **Gender** | **Upper gastrointestial involvement** | **Perianal disease** | **Complications** | **C-reactive protein** | **Erythrocyte sedimentation rate** | **Fecal calprotectin** | **Fecal occult blood** |
| --- | --- | --- | --- | --- | --- | --- | --- | --- | --- | --- | --- | --- | --- | --- | --- |
| 100M | CD | Stricture | Ileum | without | 199 | Active | 26 | Male | without | without | without | 0.46 | 30 | >60 | positive |
| 108M | UC | Chronic relapsing | Extensive colitis | 10 | without | Active | 27 | Female | without | No data | without | 0.38 | 6 | >60 | positive |
| 110M | CD | Non-stricturing non-penetrating | Ileocolon | without | 244 | Active | 23 | Male | without | with | without | 4.16 | 58 | 37.5 | negative |
| 111M | UC | Chronic relapsing | Extensive colitis | 11 | without | Active | 52 | Male | without | without | without | 7.11 | 62 | 37.5 | positive |
| 113M | UC | Chronic relapsing | Extensive colitis | 3 | without | Active | 59 | Male | without | without | without | 0.9 | 23 | >60 | positive |
| 114M | UC | Chronic relapsing | Extensive colitis | 8 | without | Active | 61 | Female | without | without | stricture | 8.33 | 23 | >60 | positive |
| 115M | CD | Stricture | Ileocolon | without | 367 | Active | 18 | Male | without | without | stricture | 6 | 36 | >60 | positive |
| 116M | UC | Chronic relapsing | Extensive colitis | 9 | without | Active | 39 | Female | without | without | carcinogenesis | 1.28 | 19 | 37.5 | positive |
| 118M | UC | Chronic relapsing | Extensive colitis | 1 | without | Remission | 66 | Male | without | without | without | 0.12 | 17 | 37.5 | negative |
| 130M | UC | Chronic relapsing | Extensive colitis | 9 | without | Active | 35 | Female | without | without | without | 0.38 | 16 | 37.5 | positive |
| 131M | UC | Chronic relapsing | Left-sided colitis | 8 | without | Active | 56 | Male | without | without | without | 0 | 0 | 0 | No data |
| 133M | UC | Chronic relapsing | Extensive colitis | 2 | without | Remission | 49 | Male | without | without | without | 0.1 | 6 | 15 | negative |
| 134M | UC | Chronic relapsing | Extensive colitis | 9 | without | Active | 35 | Female | without | without | Extraintestinal manifestations | 1.2 | 13 | 37.5 | positive |
| 135M | UC | Initial onset | Extensive colitis | 9 | without | Active | 37 | Male | without | without | without | 0.38 | 11 | 37.5 | positive |
| 137M | CD | Non-stricturing non-penetrating | Ileocolon | without | 326 | Active | 22 | Male | without | with | without | 13.8 | 83 | 37.5 | negative |
| 138M | CD | Penetrating | Ileocolon | without | 184 | Active | 27 | Male | without | with | penetrating | 7.69 | 33 | >60 | negative |
| 139M | UC | Chronic relapsing | Extensive colitis | 11 | without | Active | 27 | Male | without | without | without | 7.92 | 73 | 37.5 | positive |
| 13M | UC | Chronic relapsing | Left-sided colitis | 4 | without | Active | 46 | Male | without | without | without | 0.17 | 2 | 0 | positive |
| 146M | CD | Non-stricturing non-penetrating | Ileocolon | without | 115 | Remission | 18 | Male | with | with | without | 0.1 | 2 | 15 | negative |
| 149M | UC | Chronic relapsing | Extensive colitis | 12 | without | Active | 56 | Female | without | without | without | 1.19 | 19 | >60 | positive |
| 150M | UC | Chronic relapsing | Extensive colitis | 7 | without | Active | 73 | Female | without | without | carcinogenesis | 0.77 | 31 | >60 | negative |
| 151M | UC | Initial onset | Rectum | 5 | without | Active | 50 | Male | without | without | without | 0.44 | 18 | >60 | positive |
| 153M | UC | Non-stricturing non-penetrating | Ileocolon | without | without | Remission | 25 | Male | without | with | without | 0.65 | 21 | >60 | negative |
| 154M | UC | Chronic relapsing | Extensive colitis | 10 | without | Active | 66 | Male | with | without | without | 1.26 | 19 | >60 | positive |
| 155M | UC | Stricture | Ileocolon | without | without | Remission | 40 | Male | without | without | stricture | 4.71 | 21 | 37.5 | negative |
| 158M | CD | Stricture | Ileum | without | 6 | Remission | 38 | Male | with | without | stricture | 0.28 | 1 | 37.5 | positive |
| 159M | UC | Initial onset | Extensive colitis | 3 | without | Active | 73 | Female | without | without | without | 0.85 | 27 | 37.5 | positive |
| 160M | UC | Chronic relapsing | Extensive colitis | 4 | without | Active | 54 | Female | without | without | without | 0.11 | 2 | >60 | positive |
| 161M | CD | Non-stricturing non-penetrating | Ileocolon | without | 28 | Remission | 34 | Male | without | with | without | 0.18 | 3 | 37.5 | positive |
| 162M | CD | Stricture | Ileocolon | without | 169 | Active | 53 | Male | without | without | without | 1.88 | 49 | >60 | positive |
| 163M | CD | Penetrating | Ileocolon | without | 60 | Remission | 37 | Male | without | with | penetrating | 0.77 | 2 | >60 | negative |
| 164M | CD | Non-stricturing non-penetrating | Ileum | without | 8 | Remission | 65 | Male | without | without | without | 0.97 | 19 | >60 | negative |
| 166M | UC | Chronic relapsing | Left-sided colitis | 12 | without | Active | 39 | Female | without | without | without | 3.7 | 39 | 37.5 | positive |
| 167M | CD | Penetrating | Ileocolon | without | 35 | Remission | 28 | Male | without | without | surgery | 0.4 | 6 | 37.5 | negative |
| 168M | CD | Penetrating | Ileocolon | without | 236 | Active | 21 | Male | with | with | surgery | 7.83 | 23 | 37.5 | positive |
| 16M | CD | Stricture | Ileocolon | without | 268 | Active | 22 | Male | without | with | without | 6.51 | 16 | 0 | positive |
| 170M | CD | Stricture | Colon | without | 92 | Remission | 65 | Male | without | with | stricture | 5.01 | 10 | >60 | positive |
| 171M | CD | Non-stricturing non-penetrating | Ileocolon | without | 0 | Remission | 22 | Male | without | with | without | 0.25 | 2 | 37.5 | negative |
| 17M | UC | Chronic relapsing | Left-sided colitis | 4 | without | Active | 31 | Female | without | without | without | 0.12 | 3 | 0 | positive |
| 185M | UC | Chronic relapsing | Rectum | 8 | without | Active | 45 | Male | without | without | without | 0.4 | 7 | 37.5 | positive |
| 190M | CD | Penetrating | Ileocolon | without | 108 | Remission | 23 | Male | without | with | penetrating | 9.22 | 44 | >60 | positive |
| 191M | UC | Chronic relapsing | Extensive colitis | without | without | Active | 26 | Female | without | without | without | 0.47 | 19 | 36 | positive |
| 192M | CD | Stricture | Ileum | without | 68 | Remission | 31 | Female | with | with | without | 0.1 | 6 | >60 | positive |
| 193M | CD | Non-stricturing non-penetrating | Ileocolon | without | 42 | Remission | 30 | Female | without | with | without | 0.27 | 14 | >60 | negative |
| 194M | UC | Initial onset | Extensive colitis | 11 | without | Active | 64 | Female | without | without | carcinogenesis | 1.67 | 10 | 13 | negative |
| 195M | IBD | Initial onset | Extensive colitis | without | without | Active | 34 | Female | without | without | without | 3.07 | 7 | >60 | positive |
| 196M | CD | Stricture | Ileocolon | without | 194 | Active | 57 | Female | without | without | without | 0.35 | 2 | >60 | positive |
| 197M | UC | Chronic relapsing | Extensive colitis | 11 | without | Active | 46 | Male | without | without | without | 0.24 | 28 | 17 | positive |
| 198M | CD | Non-stricturing non-penetrating | Ileocolon | without | 30 | Remission | 28 | Male | without | with | without | 0.64 | 2 | >60 | positive |
| 200M | UC | Non-stricturing non-penetrating | Colon | without | without | Remission | 22 | Male | without | without | without | 2.36 | 34 | >60 | positive |
| 201M | UC | Chronic relapsing | Left-sided colitis | 5 | without | Active | 29 | Male | without | without | without | 0.32 | 4 | 20 | positive |
| 206M | CD | Non-stricturing non-penetrating | Ileocolon | without | 19 | Remission | 30 | Male | without | without | without | 0.3 | 14 | 14 | negative |
| 210M | UC | Chronic relapsing | Extensive colitis | 8 | without | Active | 52 | Female | without | without | surgery | 1.03 | 49 | 30 | positive |
| 211M | CD | Stricture | Ileocolon | without | 60 | Remission | 28 | Male | without | without | surgery | 0.14 | 1 | 10 | negative |
| 214M | CD | Non-stricturing non-penetrating | Ileocolon | without | 224 | Active | 29 | Male | without | without | without | 0 | 0 | 0 | negative |
| 215M | UC | Chronic relapsing | Extensive colitis | without | without | Active | 40 | Male | without | without | without | 0 | 0 | 0 | negative |
| 216M | CD | without | Ileocolon | without | 149 | Remission | 29 | Female | without | without | surgery | 0.23 | 23 | 22 | negative |
| 217M | CD | Penetrating | Ileum | without | 60 | Remission | 33 | Male | without | with | penetrating | 1.21 | 2 | 44 | negative |
| 218M | CD | Stricture | Ileum | without | 40 | Remission | 20 | Male | without | without | stricture | 0.17 | 1 | 25 | negative |
| 222M | IBD | Stricture | Ileocolon | without | without | Active | 32 | Male | without | without | stricture | 3.11 | 39 | >60 | positive |
| 223M | UC | Chronic relapsing | Extensive colitis | 2 | without | Remission | 38 | Male | without | without | Extraintestinal manifestations | 0.26 | 1 | 48 | positive |
| 224M | CD | Penetrating | Ileocolon | without | 243 | Active | 24 | Female | without | without | penetrating | 2.39 | 41 | 10 | negative |
| 225M | CD | Non-stricturing non-penetrating | Colon | without | 174 | Active | 38 | Female | without | without | Extraintestinal manifestations | 0.14 | 9 | >60 | negative |
| 232M | UC | Initial onset | Extensive colitis | 11 | without | Active | 39 | Male | without | with | without | 0.7 | 14 | >60 | positive |
| 236M | UC | Chronic relapsing | Extensive colitis | 6 | without | Active | 17 | Male | without | without | without | 0.25 | 2 | 31 | positive |
| 239M | UC | Chronic relapsing | Extensive colitis | 8 | without | Active | 46 | Male | without | without | without | 0.2 | 8 | 43 | positive |
| 23M | UC | without | without | 5 | without | Active | 26 | Female | No data | No data | without | 0.23 | 0 | 0 | positive |
| 24M | UC | Chronic relapsing | Extensive colitis | 4 | without | Active | 69 | Male | without | without | without | 0.13 | 0 | 0 | positive |
| 250M | UC | Chronic relapsing | Extensive colitis | 9 | without | Active | 54 | Female | without | without | without | 1.98 | 0 | 0 | No data |
| 255M | IBD | Penetrating | Ileocolon | without | without | Remission | 36 | Male | without | with | surgery | 0.1 | 3 | 15 | negative |
| 257M | UC | Initial onset | Extensive colitis | 5 | without | Active | 49 | Male | without | without | without | 0.44 | 28 | 45 | negative |
| 2M | UC | Chronic relapsing | Rectum | 5 | without | Active | 38 | Male | without | without | without | 0.23 | 2 | 0 | positive |
| 32M | UC | Chronic relapsing | Extensive colitis | 3 | without | Active | 35 | Male | No data | with | without | 0.91 | 7 | >60 | positive |
| 33M | CD | Non-stricturing non-penetrating | Colon | without | 20 | Remission | 55 | Female | without | without | without | 0.32 | 2 | 0 | negative |
| 34M | CD | Stricture | Ileocolon | without | 47 | Remission | 40 | Male | without | with | without | 0.41 | 14 | >60 | positive |
| 35M | CD | Non-stricturing non-penetrating | Colon | without | 74 | Remission | 18 | Female | without | with | without | 0.15 | 6 | >60 | positive |
| 41M | UC | Chronic relapsing | Extensive colitis | 2 | without | Remission | 53 | Male | without | without | without | 3.49 | 59 | >60 | positive |
| 42M | CD | Stricture | Ileocolon | without | 139 | Remission | 28 | Male | with | with | without | 0.11 | 5 | 37.5 | negative |
| 43M | CD | Non-stricturing non-penetrating | Ileum | without | 67 | Remission | 25 | Male | without | without | without | 0.12 | 2 | 15 | negative |
| 48M | UC | Chronic relapsing | Extensive colitis | 12 | without | Active | 22 | Male | without | without | without | 0.4 | 1 | 37.5 | positive |
| 49M | UC | Initial onset | Extensive colitis | 11 | without | Active | 50 | Male | without | without | without | 3.56 | 1 | >60 | positive |
| 4M | CD | Non-stricturing non-penetrating | Colon | without | 70 | Active | 23 | Male | without | with | No data | 3.85 | 17 | >60 | positive |
| 52M | CD | Stricture | Ileum | without | 69 | Remission | 29 | Male | without | with | without | 0.91 | 19 | >60 | positive |
| 53M | CD | Stricture | Ileum | without | 91 | Remission | 53 | Male | without | without | stricture | 0.2 | 3 | 37.5 | positive |
| 54M | CD | Non-stricturing non-penetrating | Ileum | without | 20 | Remission | 26 | Male | without | with | without | 0.12 | 1 | 37.5 | positive |
| 56M | CD | Stricture | Colon | without | 63 | Remission | 37 | Female | without | without | surgery | 0.23 | 6 | 37.5 | negative |
| 57M | CD | Non-stricturing non-penetrating | Ileocolon | without | 36 | Remission | 34 | Male | without | with | without | 0.51 | 5 | 37.5 | negative |
| 59M | UC | Initial onset | Extensive colitis | 2 | without | Remission | 28 | Male | without | without | without | 0.12 | 4 | 37.5 | positive |
| 65M | CD | Non-stricturing non-penetrating | Ileocolon | without | without | Remission | 61 | Female | with | without | without | 0.37 | 6 | >60 | positive |
| 66M | UC | Chronic relapsing | Extensive colitis | 11 | without | Active | 37 | Female | without | with | without | 0.43 | 17 | >60 | positive |
| 67M | UC | Chronic relapsing | Left-sided colitis | 5 | without | Active | 61 | Female | without | without | carcinogenesis | 0.41 | 13 | 37.5 | negative |
| 68M | CD | Stricture | Ileocolon | without | 41 | Remission | 32 | Female | without | with | stricture | 0.14 | 10 | 15 | negative |
| 70M | IBD | Non-stricturing non-penetrating | Ileocolon | without | without | Remission | 28 | Male | with | without | stricture | 0.1 | 1 | >60 | positive |
| 71M | UC | Chronic relapsing | Extensive colitis | 2 | without | Remission | 28 | Male | without | without | Extraintestinal manifestations | 0.1 | 1 | 37.5 | negative |
| 76M | CD | Stricture | Colon | without | 95 | Remission | 28 | Female | without | without | stricture | 0.13 | 12 | 37.5 | positive |
| 78M | UC | Chronic relapsing | Left-sided colitis | 6 | without | Active | 80 | Male | without | without | carcinogenesis | 0.45 | 11 | 37.5 | positive |
| 79M | UC | Chronic relapsing | Extensive colitis | 8 | without | Active | 45 | Male | without | without | surgery | 2.72 | 34 | >60 | positive |
| 82M | UC | Chronic relapsing | Left-sided colitis | 3 | without | Remission | 40 | Male | without | with | without | 0.1 | 3 | >60 | positive |
| 83M | CD | Stricture | Ileum | without | 30 | Remission | 57 | Male | without | without | carcinogenesis | 0.2 | 2 | 37.5 | negative |
| 84M | CD | Non-stricturing non-penetrating | Ileocolon | without | 290 | Active | 18 | Male | without | with | without | 3.05 | 15 | >60 | positive |
| 85M | CD | Stricture | Ileocolon | without | 24 | Remission | 30 | Male | with | with | stricture | 3.26 | 36 | >60 | positive |
| 87M | CD | Stricture | Ileocolon | without | 61.5 | Remission | 23 | Male | with | without | stricture | 0.83 | 2 | >60 | negative |
| 88M | UC | Initial onset | Extensive colitis | 7 | without | Active | 21 | Female | without | without | without | 0.59 | 19 | 37.5 | positive |
| 89M | CD | Penetrating | Ileocolon | without | 48 | Remission | 56 | Female | with | without | penetrating | 0.13 | 9 | >60 | negative |
| 94M | CD | Stricture | Colon | without | 166 | Active | 21 | Female | without | with | stricture | 12.8 | 89 | >60 | positive |
| 98M | CD | Non-stricturing non-penetrating | Ileocolon | without | 36 | Remission | 22 | Male | without | with | without | 0.15 | 4 | 37.5 | negative |
| 9M | UC | Chronic relapsing | Extensive colitis | 5 | without | Active | 30 | Male | without | without | without | 0.11 | 1 | 0 | negative |

# CDAI (Crohn's Disease Activity Index): This is a research tool used to quantify the symptoms and features of Crohn's Disease. It helps in assessing disease severity and is commonly used in clinical trials to determine the efficacy of treatments.

# Mayo Score: This is a scoring system used to assess the severity of ulcerative colitis. It includes subscores for stool frequency, rectal bleeding, endoscopic findings, and physician's global assessment, providing a comprehensive overview of disease activity.

**Supplementary Table 6** The SHAP value (above 0) of each feature in metagenomic and clinic data-based classification model.

| The microbial species | SHAP values |
| --- | --- |
| *Clostridium perfringens* | 1.0515 |
| Perianal disease | 0.5330 |
| *Streptococcus mitis* | 0.5325 |
| *Clostridium innocuum* | 0.4055 |
| Fecal occult blood | 0.2937 |
| *Escherichia coli* | 0.2855 |
| *Intestinibacter bartlettii* | 0.2353 |
| ESR | 0.2273 |
| *Streptococcus infantis* | 0.1871 |
| *Parabacteroides distasonis* | 0.1746 |
| *Enterococcus faecium* | 0.1613 |
| *Clostridium spiroforme* | 0.1591 |
| *Lactobacillus mucosae* | 0.1527 |
| *Ruminococcus gnavus* | 0.1414 |
| *Gordonibacter pamelaeae* | 0.1252 |
| *Blautia producta* | 0.1177 |
| *Actinomyces sp oral taxon* | 0.1079 |
| *Bifidobacterium longum* | 0.0958 |
| *Bacteroides fragilis* | 0.0909 |
| *Veillonella atypica* | 0.0815 |
| Complications | 0.0763 |
| *Fusobacterium ulcerans* | 0.0726 |
| *Bacteroides cellulosilyticus* | 0.0644 |
| Fecal calprotectin | 0.0627 |
| *Clostridium clostridioforme* | 0.0609 |
| *Roseburia inulinivorans* | 0.0594 |
| *Erysipelatoclostridium ramosum* | 0.0578 |
| *Bacteroides ovatus* | 0.0518 |
| *Blautia wexlerae* | 0.0483 |
| *Actinomyces odontolyticus* | 0.0475 |
| *Blautia hansenii* | 0.0474 |
| *Hungatella hathewayi* | 0.0432 |
| *Gemella sanguinis* | 0.0377 |
| *Streptococcus salivarius* | 0.0346 |
| *Blautia obeum* | 0.0329 |
| *Flavonifractor plautii* | 0.0305 |
| *Streptococcus sanguinis* | 0.0283 |
| *Eubacterium hallii* | 0.0280 |
| *Agathobaculum butyriciproducens* | 0.0277 |
| *Solobacterium moorei* | 0.0267 |
| C reactive protein | 0.0211 |
| *Bacteroides stercoris* | 0.0210 |
| *Eggerthella lenta* | 0.0198 |
| *Klebsiella oxytoca* | 0.0164 |
| *Romboutsia ilealis* | 0.0124 |
| *Phascolarctobacterium faecium* | 0.0117 |
| *Actinomyces sp HMSC035G02* | 0.0115 |
| *Veillonella sp T11011* | 0.0115 |
| *Clostridium bolteae* | 0.0100 |
| *Actinomyces sp ICM47* | 0.0092 |
| *Veillonella parvula* | 0.0051 |
